# USP9X-mediated deubiquitination of Raptor contributes to autophagy impairment and memory deficits in P301S mice

**DOI:** 10.1186/s12964-024-01872-8

**Published:** 2024-10-24

**Authors:** Siyi Zheng, Jiahui Zhu, Cailin Wang, Yanqing Wu, Shangqi Sun, Hongxiu Guo, Yanmin Chang, Rong Ma, Gang Li

**Affiliations:** 1 Department of Neurology, Union Hospital, Tongji Medical College, Huazhong University of Science and Technology, Wuhan, 430022 China; 2 Department of Neurology, Wuhan Fourth Hospital, Wuhan, 430033 China; 3 Department of Pharmacology, School of Basic Medicine, Tongji Medical College, Huazhong University of Science and Technology, Wuhan, 430030 China

**Keywords:** Tau, Raptor, USP9X, Autophagy, Alzheimer’s disease

## Abstract

**Background:**

Tauopathies, including Alzheimer’s disease, are characterized by the pathological aggregation of tau protein, which is strongly linked to dysregulation of the autophagy-lysosomal degradation pathway. However, therapeutic strategies targeting this pathway remain limited.

**Methods:**

We used both in vitro and in vivo models to investigate the role of Raptor in tau pathology. Knockdown of Raptor was performed to assess its impact on mTORC1 activation, autophagy, and tau accumulation. The relationship between USP9X and Raptor was also examined. Pharmacological inhibition of USP9X with WP1130 was employed to further confirm the involvement of the USP9X-Raptor-mTORC1 axis in tau degradation.

**Results:**

Elevated Raptor levels in the hippocampus of P301S mice led to hyperactivation of mTORC1, impairing autophagy flux. Knockdown of Raptor effectively suppressed mTORC1 activation, promoted autophagy, and mitigated the accumulation of tau and its phosphorylated isoforms. This reduction in tau pathology was accompanied by decreased neuronal loss in the hippocampus, amelioration of synaptic damage, and improvement in cognitive function. The increased Raptor protein observed in the hippocampus of P301S mice was likely attributable to elevated USP9X content, which enhanced Raptor deubiquitination and protected it from proteasomal degradation. Pharmacological inhibition of USP9X with WP1130 in vitro effectively suppressed Raptor, promoted autophagy, and accelerated the degradation of tau and phosphorylated tau.

**Conclusions:**

Our findings highlight Raptor and USP9X as promising molecular targets for therapeutic intervention in tauopathies. Targeting the USP9X-Raptor-mTORC1 axis may provide a novel strategy for promoting autophagy and mitigating tau pathology in Alzheimer’s disease and other tauopathies.

**Graphical Abstract:**

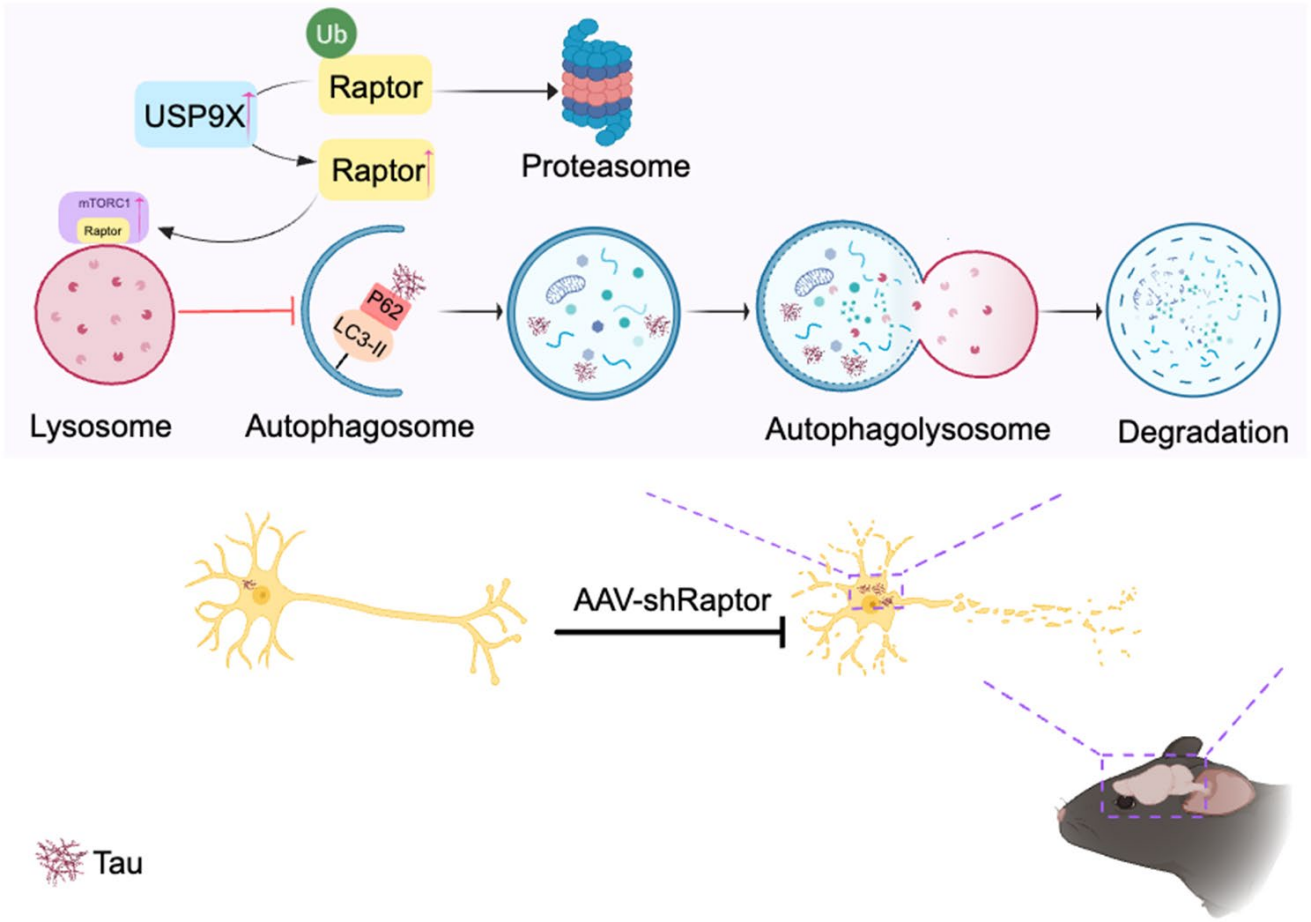

**Supplementary Information:**

The online version contains supplementary material available at 10.1186/s12964-024-01872-8.

## Introduction

The global population is aging rapidly, leading to a projected surge in the number of individuals living with dementia, from 55 million in 2019 to 139 million by 2050, as highlighted by the World Alzheimer Report 2023. Two recently FDA-approved drugs, aducanumab and lecanemab, have shown potential to slow cognitive and functional decline in early Alzheimer’s disease by targeting beta-amyloid plaques. However, they are not suitable for everyone and do not offer a cure [1]. The accumulation of Aβ plaques and neurofibrillary tangles, composed of aggregated Aβ and tau proteins respectively, are hallmark pathological features of AD [2]. Consequently, strategies aimed at promoting the degradation of these proteins represent a promising therapeutic avenue. Among the cellular degradation pathways, the ubiquitin-proteasome system and autophagy-lysosome pathway are particularly relevant. The autophagy pathway, in particular, has garnered increasing attention for its potential role in AD pathogenesis [3, 4]. This has led to a growing interest in exploring the therapeutic potential of enhancing autophagy as a strategy for treating AD [5].

The mechanistic target of rapamycin (mTOR) is a central regulator of cellular metabolism and signaling. It belongs to the phosphoinositide 3-kinase (PI3K) family and exhibits dual kinase activity towards serine/threonine and tyrosine residues [6]. mTOR forms distinct complexes, including mTORC1 and mTORC2, which play crucial roles in modulating synaptic plasticity and memory formation [7, 8]. Notably, mTORC1 is a key suppressor of autophagy [9], a cellular process implicated in AD pathogenesis. While evidence suggests upregulated mTORC1 activity in the brains of AD patients and mouse models, potentially contributing to impaired autophagy [10–13], contradictory findings have also been reported. For instance, reduced phosphorylation of proteins within the mTORC1 signaling pathway has been observed in Aβ-treated cultured neurons [14], young Tg2576 mice [15] and aged APP/PS1 mice [16], highlighting the complexity of mTORC1 signaling in AD. Interestingly, rapamycin, an mTORC1 inhibitor, has been shown to reduce amyloid secretion through moderate autophagy induction in neurons, both in APP/PSEN1 mice and primary neuronal cultures [17, 18]. Furthermore, pharmacological inhibition of mTORC1 has been shown to promote autophagy, reduce tau protein levels, and exert neuroprotective effects in both in vitro and in vivo models [19, 20].

Raptor, an essential component of mTORC1 that governs complex assembly, localization, stability, and substrate recruitment [6], has been found at elevated levels in the hippocampus of individuals with advanced AD, suggesting a potential link between Raptor dysregulation and AD pathogenesis [10]. Raptor knockdown has demonstrated promising therapeutic effects in other neurological disorders. In fragile X syndrome, Raptor depletion suppressed mTORC1 activity, enhanced autophagy, and ameliorated synaptic and cognitive impairments [21, 22]. Similarly, in tuberous sclerosis complex, Raptor downregulation rescued neuronal phenotypes in mouse models [23]. However, the impact of Raptor on tau pathology remains largely unexplored. Therefore, investigating whether Raptor knockdown can promote tau protein degradation by inhibiting mTORC1 activation and inducing autophagy is a pressing research priority.

The gonosomal gene USP9 (ubiquitin specific peptidase 9) exists in two forms, USP9Y on the Y chromosome and USP9X on the X chromosome, with the latter escaping X-inactivation in humans. The gene encodes a deubiquitinase that removes ubiquitin moieties from specific proteins, thereby preventing their proteasomal degradation [24]. USP9 has been shown to influence the phosphorylation of microtubule-associated protein tau (MAPT) by deubiquitinating the kinase MARK4, which directly phosphorylates MAPT, and by deubiquitinating α-synuclein (SNCA), which mediates the connection between glycogen synthase kinase 3β (GSK3B) and its phosphorylation target MAPT [25]. Previous studies have demonstrated that USP9 knockdown significantly reduces MAPT expression in a DU145 cell culture model, as well as the expression of its orthologs in zebrafish [25]. Furthermore, USP9X deubiquitinates Raptor, thereby influencing mTOR signaling and potentially modulating autophagy. Notably, USP9X’s impact on mTORC1 appears cell context-dependent, with knockout reducing signaling in ReNcell VM cells while knockdown increases activity in C2C12 myoblasts [26]. Despite these findings, the role of USP9X in tau pathology remains largely unexplored. Given the established links between USP9X, autophagy, and tau phosphorylation, investigating the potential role of USP9X in regulating tau protein levels through Raptor deubiquitination in the context of tauopathy is of paramount importance.

Here, we demonstrate that Raptor knockdown in the P301S mouse model significantly ameliorates tau pathology. This effect is mediated by suppression of mTORC1 hyperactivation, leading to enhanced autophagy and subsequent reductions in tau and phosphorylated tau accumulation. Furthermore, Raptor knockdown mitigates neuronal loss, synaptic damage, and ultimately leads to cognitive improvement. Mechanistically, we show that elevated Raptor protein levels in P301S mice are likely attributed to increased USP9X content, which enhances Raptor deubiquitination, thereby protecting it from proteasomal degradation. Our findings highlight Raptor and USP9X as potential therapeutic targets for tauopathy, offering promising avenues for novel treatment strategies.

## Results

### Raptor expression and mTORC1 signaling are altered in P301S mice

**Fig. 1:**
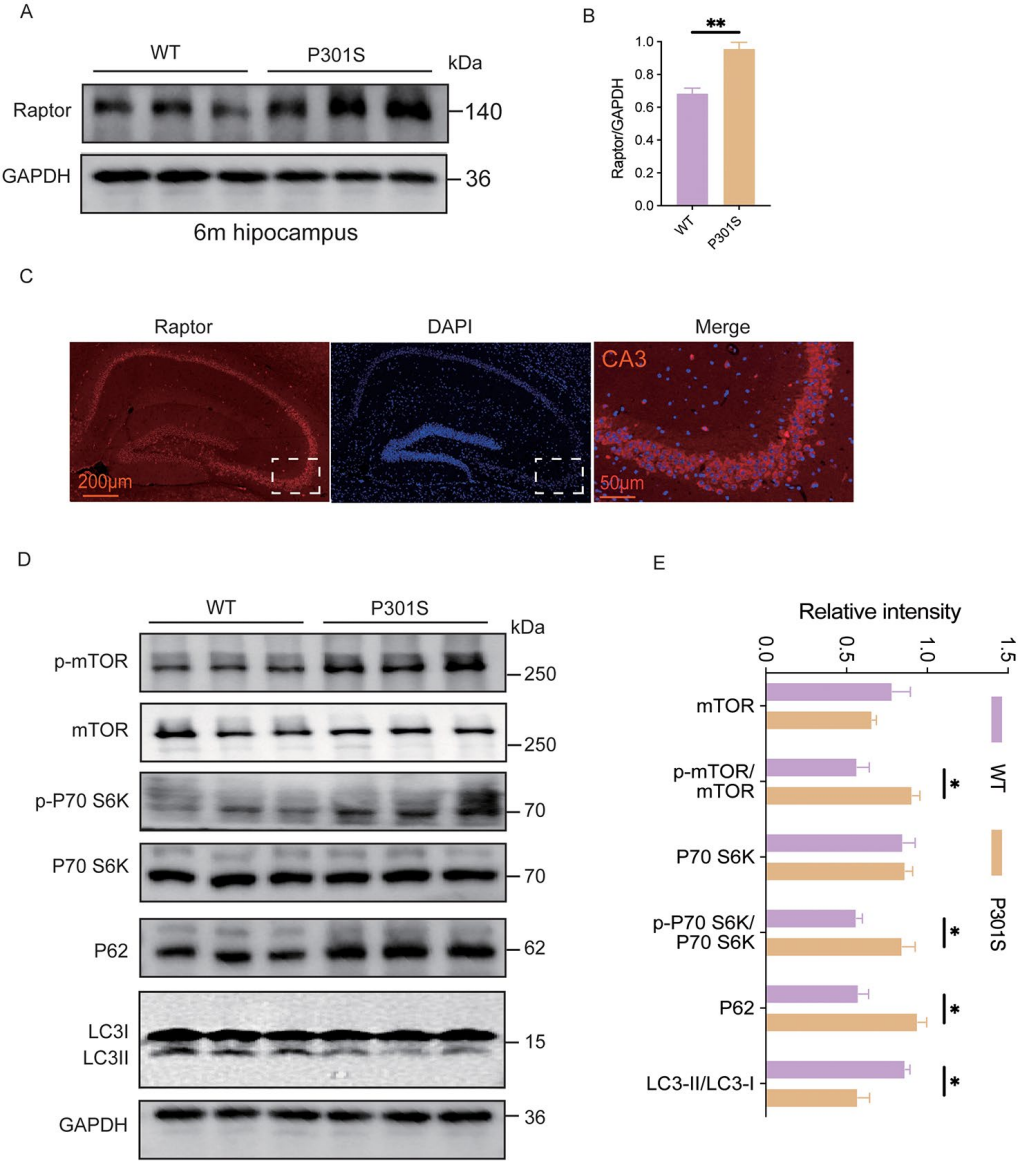
Raptor expression and mTOR signaling were altered in P301S mice. (**A**, **B**) Representative Western blots and quantification of Raptor protein levels in the hippocampus of 6-month-old P301S mice and WT littermates. *n* = 3 mice per group. (**C**) Representative immunofluorescent images showing Raptor expression (red) in the hippocampus of 7-month-old P301S mice. Cell nuclei are stained with DAPI (blue). (**D**, **E**) Representative western blots and quantification of mTORC1 signaling pathway components in the hippocampus of 6-month-old P301S and WT mice. Proteins analyzed include mTOR, p-mTOR, P70 S6K, p-P70 S6K, P62, and LC3 expression. *n* = 3 mice per group. Unpaired t-tests were used to determine statistical significance. All data were presented as mean ± SEM. ns indicates no statistically significant difference, *p < 0.05, **p < 0.01, ***p < 0.001, ****p < 0.0001

Previous studies have demonstrated that mTORC1 activity is elevated in the brains of individuals with AD, characterized by significantly increased levels of Raptor in the hippocampus during severe stages of the disease [10]. Further supporting this observation, analysis of the GSE5281 dataset revealed an upward trend in Raptor gene expression in postmortem superior frontal gyrus samples from AD patients (Fig. S1A). To investigate this in an animal model, we performed western blot analysis on hippocampal tissue from 3-, 6-, and 9-month-old P301S mice, a model of tauopathy. Our results confirmed significantly elevated Raptor levels at 6 and 9 months of age compared to age-matched wild-type (WT) controls, with no changes observed at 3 months (Fig. 1A, B; Fig. S1B-E). We also observed increased Raptor expression in the cortex of 6- and 9-month-old P301S mice compared to WT controls by western blot (Fig. S1F-I). Immunofluorescence analysis further revealed prominent Raptor signals within the hippocampus, particularly concentrated in the CA3 region (Fig. 1C). As Raptor is an essential component of the mTORC1 complex, elevated Raptor levels strongly suggest potential mTORC1 hyperactivation and a consequent impairment of autophagy. Consistent with this hypothesis, we observed increased phosphorylation of mTOR (p-mTOR) and its downstream target, P70 S6K (p-P70 S6K), in P301S mice compared to WT controls, while total mTOR and P70 S6K protein levels remained unchanged (Fig. 1D, E). These findings provide strong evidence for mTORC1 activation in the P301S model. Additionally, we observed a decreased LC3-II/LC3-I ratio and increased P62 levels in P301S mice by western blot (Fig. 1D, E). Consistent with these results, electron microscopy revealed a reduction in autophagosomes in the hippocampus of P301S mice (Fig. 3C, D), indicating impaired autophagy.

### Raptor downregulation attenuated cognitive impairments in the P301S mice

**Fig. 2:**
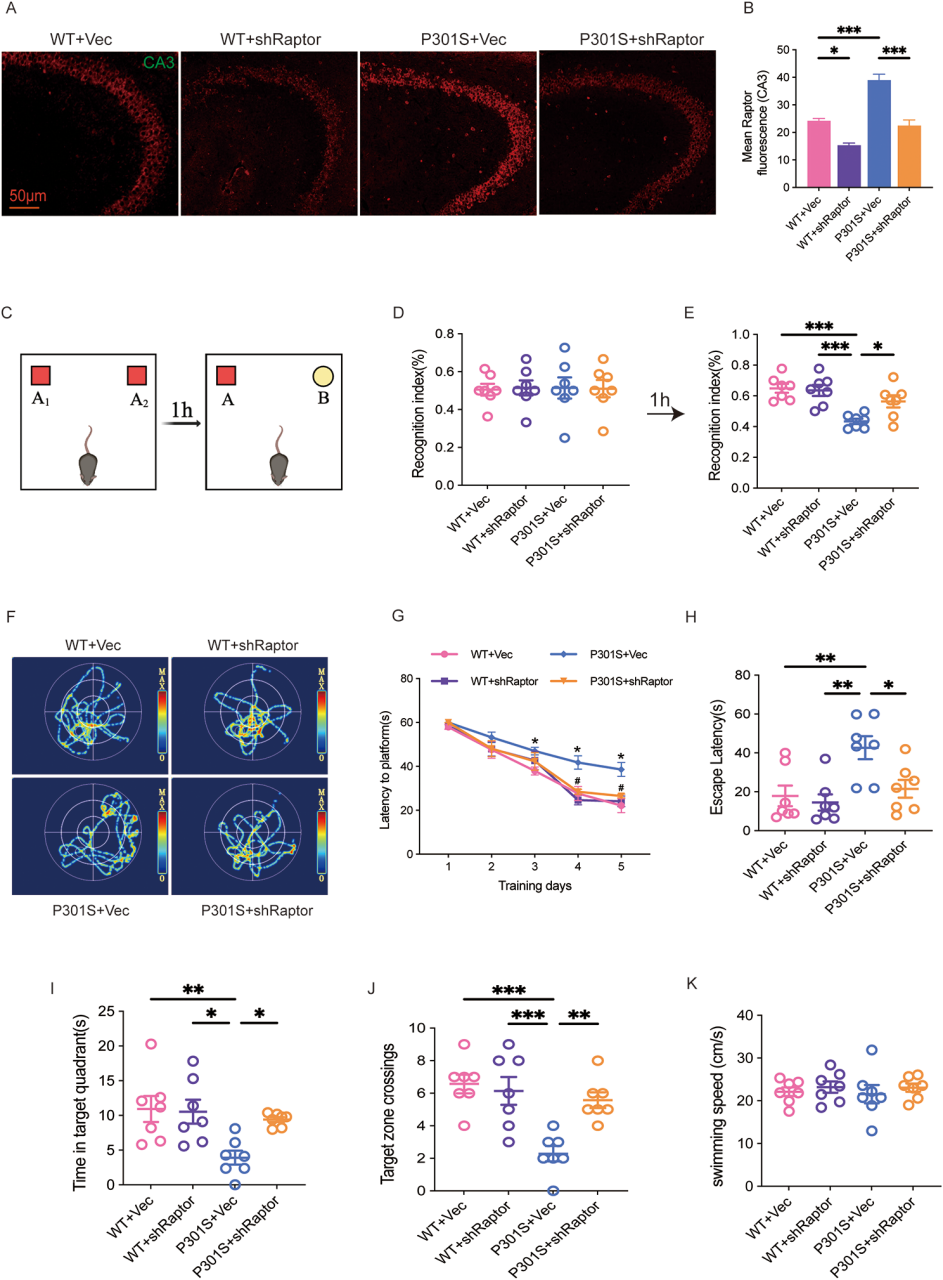
Raptor downregulation attenuated cognitive impairments in the P301S mice. (**A**, **B**) Representative immunofluorescent images showing Raptor expression in the CA3 region of the hippocampus in mice one month after stereotaxic injection. *n* = 3 mice per group. (**C**-**E**) Knockdown of Raptor enhanced recognition memory in P301S mice, as demonstrated by an increased recognition index in the NOR test. *n* = 7 mice per group. (**F**) Representative swim paths of mice during the MWM probe trial. (**G**) Raptor knockdown improved learning ability in P301S mice, as shown by a shortened latency to find the hidden platform during the training stage of the MWM test. *p < 0.05 vs. WT + Vec; ^#^*p* < 0.05 vs. P301S + Vec. (**H**-**J**) Raptor knockdown improved spatial memory in P301S mice, evidenced by a decreased latency to reach the platform location (**H**), increased time spent in the target quadrant (**I**), and a higher number of target zone crossings (**J**) during the MWM probe trial. *n* = 7 mice per group. (K) No significant differences in swimming speed were observed among the four groups during the MWM probe trial. *n* = 7 mice per group. One-way ANOVA followed by Tukey’s multiple comparisons test was used for data analysis. All data were presented as mean ± SEM. ns indicates no statistically significant difference, *p < 0.05, **p < 0.01, ***p < 0.001, ****p < 0.0001

**Fig. 3:**
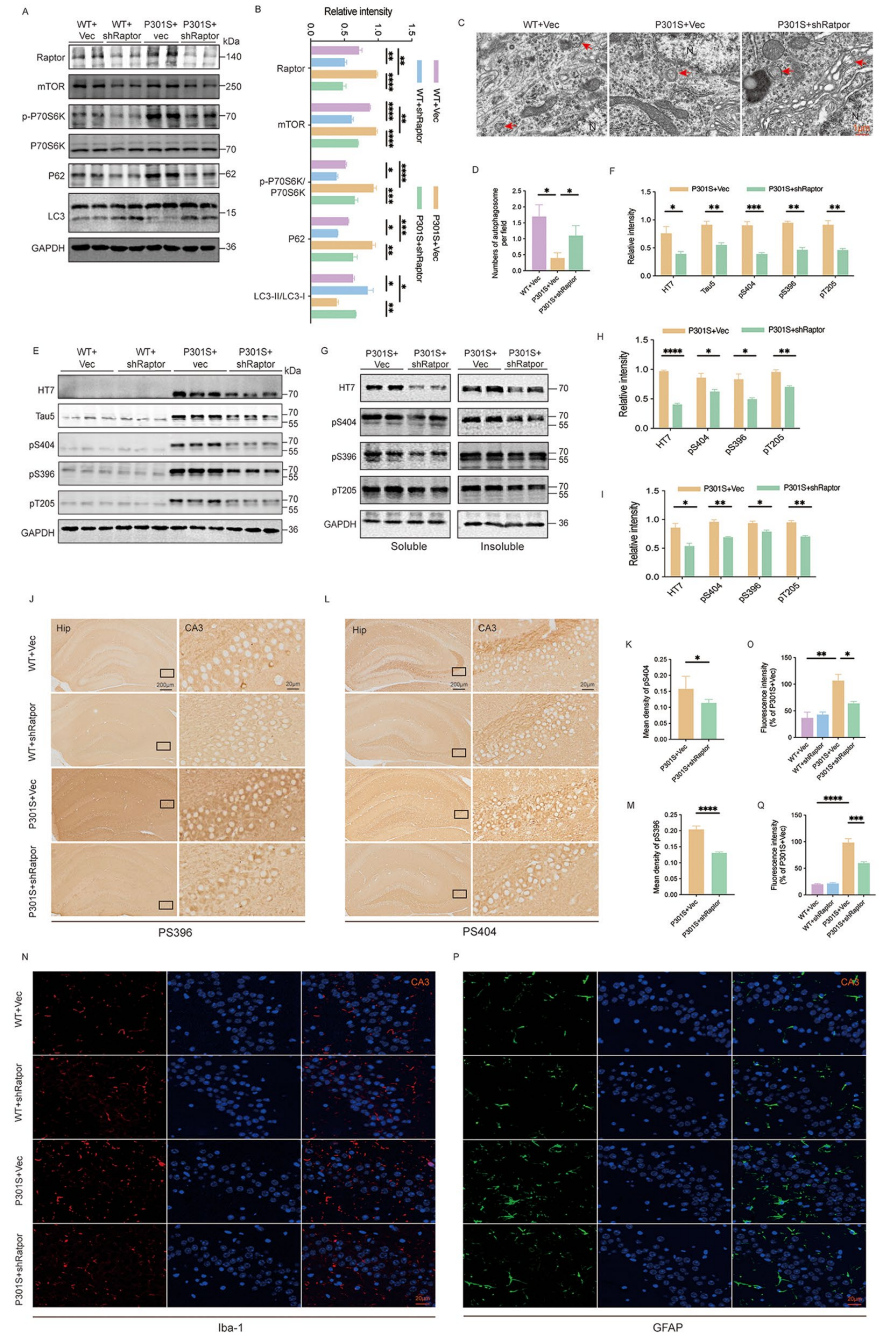
Raptor downregulation induced autophagy and reduced tau pathology in vivo. (**A**, **B**) Western blot analysis and quantitative analysis of Raptor, mTOR, p-P70 S6K, P70 S6K, P62, and LC3 expression in the hippocampus of mice. *n* = 3 mice per group. (**C**, **D**) Representative electron microscopy images showing the number of autophagosomes in the hippocampus of mice. (red arrow indicates autophagosome; N means nucleus) and quantitative analysis of autophagosome numbers. *n* = 10 images from 3 mice per group. (**E**, **F**) Western blot analysis and quantitative analysis of total tau and phosphorylated tau levels in the hippocampus of mice. *n* = 3 mice per group. (**G**) Western blot analysis of total tau and phosphorylated tau levels in sarkosyl-soluble and sarkosyl-insoluble protein fractions extracted from hippocampus. *n* = 3 mice per group. (**H**) Quantification of protein levels in the sarkosyl-soluble fraction. (**I**) Quantification of protein levels in the sarkosyl-insoluble fraction. (**J**-**M**) Representative images of immunohistochemical staining showing the effect of Raptor knockdown on tau pathology (phosphorylated tau at Ser396 or Ser404) in the CA3 area of the hippocampus and quantitative analysis of tau pathology. *n* = 5 mice per group. (**N**, **O**) Immunofluorescence detection of Iba1-positive cells in the CA3 region of the mouse hippocampus and corresponding quantification. *n* = 3 mice per group. (**P**, **Q**) Immunofluorescence detection of GFAP-positive cells in the CA3 region of the mouse hippocampus and corresponding quantification. *n* = 3 mice per group. One-way ANOVA followed by Tukey’s multiple comparisons test was used for data analysis in (**B**, **D**, **O**, **Q**). Unpaired t-tests were used for data analysis in (**F**, **H**, **I**, **K**, **M**). All data were presented as mean ± SEM. ns indicates no statistically significant difference, *p < 0.05, **p < 0.01, ***p < 0.001, ****p < 0.0001

To investigate whether Raptor knockdown could ameliorate cognitive deficits in the P301S mouse model of tauopathy, we bilaterally injected adeno-associated virus (AAV) vectors expressing either shRNA targeting Raptor (pAAV-U6-shRNA(Rptor)-CMV-EGFP-WPRE) or a control shRNA (pAAV-U6-shRNA(NC2)-CMV-EGFP-WPRE) into the hippocampal CA3 region of 6-month-old P301S mice. This age corresponds to the onset of spatial memory impairments and pronounced tau pathology in this model [27, 28]. One month after injection, learning and memory abilities were assessed using the novel object recognition (NOR) and Morris water maze (MWM) tasks (Fig. S2A). Efficient Raptor knockdown was confirmed via western blotting and immunofluorescence analyses (Figs. 2A, B; Figs. 3A, B; Fig. S2B).

In the NOR test, P301S mice exhibited significantly reduced exploration time for the novel object compared to WT controls, indicating impaired recognition memory. Raptor knockdown significantly improved novel object exploration time in P301S mice, suggesting a rescue of recognition memory deficits (Fig. 2C-E).

In the MWM test, P301S mice displayed significant learning and memory impairments compared to WT controls. This was characterized by prolonged escape latency to find the hidden platform during training (days 3–5) and reduced performance across all measures during the probe trial. These measures included increased latency to reach the previous platform location, decreased retention time in the target quadrant, and fewer platform region crossings. Notably, Raptor downregulation significantly attenuated these deficits. Treated P301S mice exhibited improved learning, demonstrated by decreased escape latency on training days 4 and 5. They also showed enhanced memory performance during the probe trial, evidenced by reduced escape latency, increased target quadrant retention time, and increased platform crossings (Fig. 2F-J). Importantly, swimming speed was comparable across all groups, confirming that observed improvements were not due to motor ability confounds (Fig. 2K). Taken together, our data demonstrated that Raptor downregulation effectively ameliorated cognitive deficits in the P301S mouse model of tauopathy.

### Raptor downregulation induced autophagy and reduced tau pathology in vivo

To investigate the underlying molecular mechanisms by which Raptor downregulation ameliorates tau-mediated cognitive impairments in P301S mice, we focused on the effects of Raptor knockdown on autophagic markers and tau pathology within the hippocampus of both WT and P301S mice. Western blot analysis revealed that Raptor downregulation significantly reduced mTORC1 activity, as evidenced by decreased mTOR levels and reduced p-P70 S6K levels without altering total P70 S6K levels. Furthermore, Raptor knockdown resulted in an increased LC3-II/LC3-I ratio and decreased P62 levels, suggesting enhanced autophagic flux (Fig. 3A, B). Electron microscopy analysis corroborated these findings, revealing an increase in autophagosome formation within the hippocampus of P301S mice following Raptor downregulation (Fig. 3C, D).

To assess the impact of Raptor knockdown on tau pathology, we examined the levels of both total tau (detected by HT7 and Tau5 antibodies) and phosphorylated tau at Ser396, Ser404, and Thr205 using western blot and immunohistochemical analyses. Raptor downregulation led to a significant reduction in both total and phosphorylated tau protein levels within the hippocampus of P301S mice (Fig. 3E, F, J-M). Further, hippocampal tissue was fractionated into sarcosyl-soluble and insoluble components. Knockdown of Raptor reduced the levels of total and phosphorylated tau in both the sarcosyl-soluble and insoluble fractions of the hippocampus in P301S mice (Fig. 3G-I).

Immunofluorescence analysis revealed a significant increase in the number of Iba1-positive microglia and GFAP-positive astrocytes in the hippocampus of the P301S group. Knockdown of Raptor resulted in a reduction of Iba1-positive and GFAP-positive cell numbers in the CA3 region of the P301S group, with no significant effects observed in the WT group (Fig. 3N-Q). Western blot analysis further supported these findings (Fig. S3A-C). These may suggest that Raptor knockdown promoted a decrease in pathological tau protein levels, thereby alleviating the reactive increase of Iba1 and GFAP.

Collectively, these data demonstrated that Raptor downregulation could promote tau degradation by activating autophagy through the inhibition of the mTORC1 signaling pathway.

### Raptor knockdown attenuates synaptic impairment and ameliorates neuronal degeneration

**Fig. 4:**
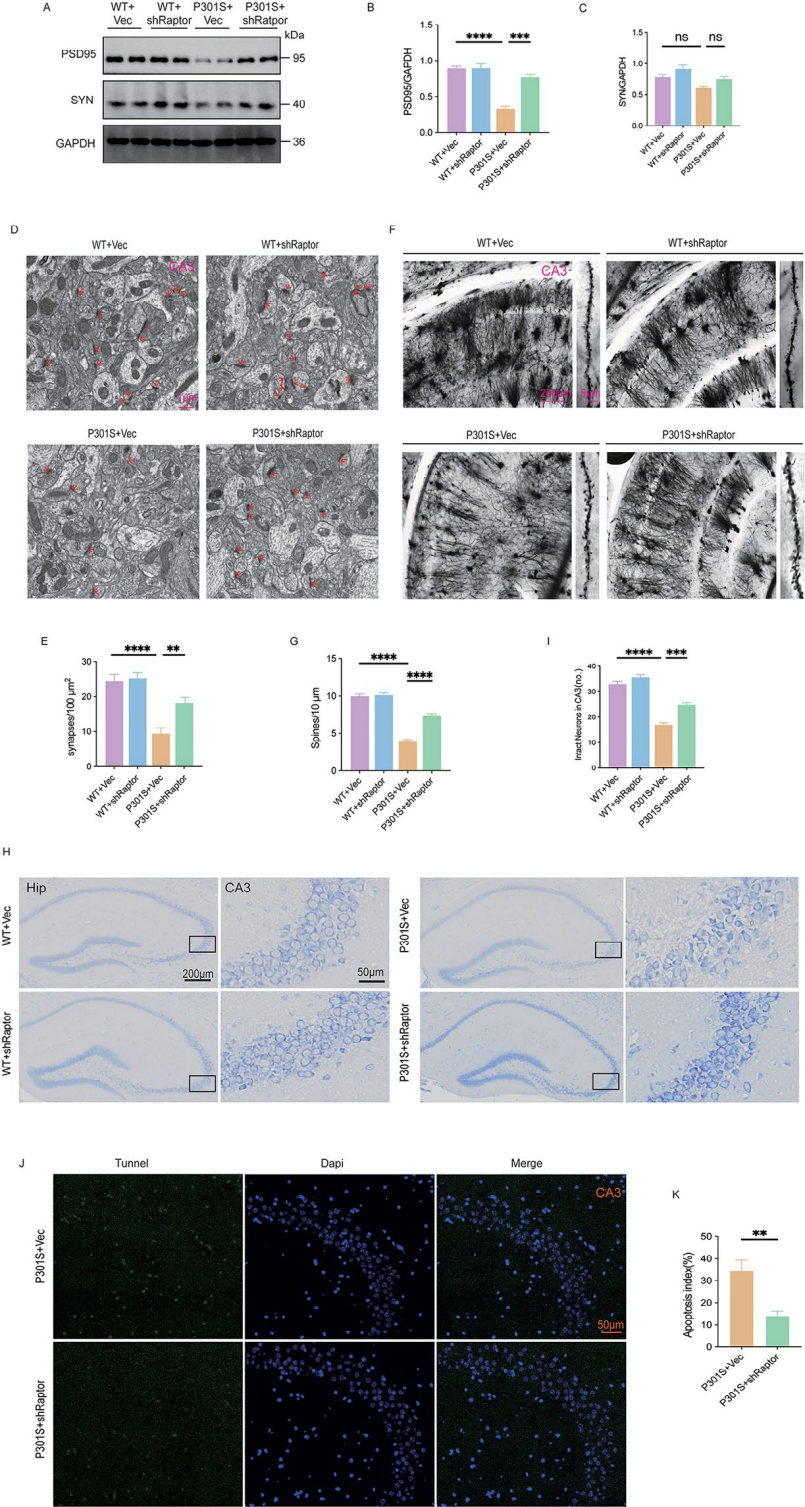
Raptor knockdown attenuated synaptic impairment and ameliorated neuronal degeneration. (**A**-**C**) Western blot analysis and quantitative analysis of PSD95 and SYN levels in the hippocampus of mice. *n* = 3 mice per group. (**D**, **E**) Electron microscopy images and quantitative analysis of synaptic density in the CA3 region of the mice hippocampus. *n* = 9 images from 3 mice per group. (**F**, **G**) Golgi staining images and quantitative analysis of dendritic spine density in the CA3 region of the mice hippocampus. *n* = 10 images from 3 mice per group. (**H**, **I**) Representative Nissl staining images of the CA3 region in the hippocampus of mice and quantitative analysis of neuronal density. *n* = 4 mice per group. (**J**) Representative images of TUNEL staining (green) in the CA3 region of the hippocampus of mice. Nuclei were counterstained with DAPI (blue). (**K**) The proportion of TUNEL-positive cells among DAPI-positive cells reflects the level of apoptosis. *n* = 5 mice per group. One-way ANOVA followed by Tukey’s multiple comparisons test was used for data analysis in (B, C, E, G, I). Unpaired t-tests were used for data analysis in (K). All data were presented as mean ± SEM. ns indicates no statistically significant difference, *p < 0.05, **p < 0.01, ***p < 0.001, ****p < 0.0001

It was reported that tau-mediated neurotoxicity is a primary driver of neuronal loss and synaptic dysfunction, hallmark neuropathological features of AD that strongly correlate with cognitive decline [29, 30]. Our study investigated the molecular mechanisms underlying the cognitive benefits of Raptor downregulation in P301S mice. Western blot analysis of hippocampal tissue revealed that Raptor downregulation selectively increased the levels of the postsynaptic protein PSD95 in P301S mice, while leaving the presynaptic protein synaptophysin (SYN) unaffected (Fig. 4A-C). Ultrastructural analysis via electron microscopy confirmed a reduction in synaptic density in the hippocampus of P301S mice. Notably, downregulation of Raptor mitigated synaptic loss in the hippocampus of P301S mice (Fig. 4D, E).

Dendritic spines, the functional protrusions on dendrites, are essential for synaptogenesis and synaptic transmission [31]. To assess the impact of Raptor downregulation on dendritic spine morphology, we employed Golgi staining to visualize and quantify dendritic spines in the CA3 region. Compared to WT mice, P301S mice exhibited sparser dendritic branching and a marked reduction in dendritic spine density. Notably, Raptor downregulation significantly attenuated this phenotype (Fig. 4F, G). To assess the impact of Raptor downregulation on neuronal integrity, we examined neuronal morphology and density in the hippocampus of WT and P301S mice using Nissl staining. In WT mice, neurons in the CA3 region exhibited a normal, organized morphology with densely packed Nissl bodies, irrespective of shRNA-mediated Raptor knockdown. In contrast, P301S mice displayed aberrant neuronal morphology and disorganization, a phenotype significantly attenuated by Raptor knockdown (Fig. 4H, I). To evaluate the effect of Raptor knockdown on cell apoptosis, we performed Tunel assays. Raptor knockdown significantly reduced the percentage of apoptotic cells in the CA3 region of the hippocampus in P301S mice (Fig. 4J, K).

Taken together, these findings demonstrated that Raptor downregulation mitigated tau-induced synaptic impairment and neuronal loss in P301S mice.

### Raptor overexpression inhibits autophagy in vitro

**Fig. 5:**
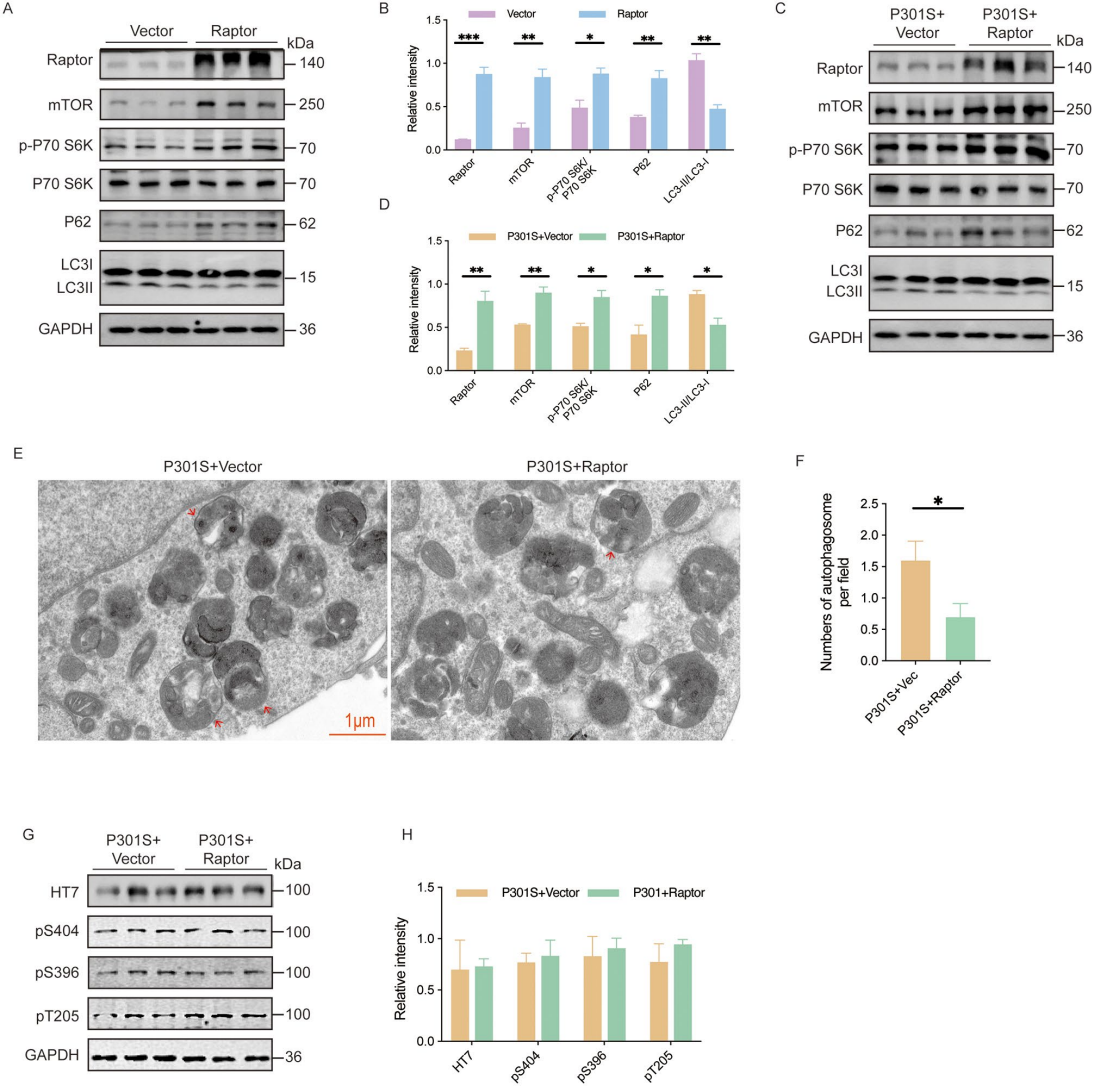
Raptor overexpression inhibited autophagy in vitro. (**A**, **B**) Western blot analysis and quantitative analysis of Raptor, mTOR, p-P70 S6K, P70 S6K, P62, and LC3 expression in HEK-293T cells 36 h after transfection with Raptor. *n* = 3 per group. (**C**, **D**) Western blot analysis and quantitative analysis of Raptor, mTOR, p-P70S6K, P70S6K, P62, and LC3 expression in HEK293/P301S cells 36 h after transfection with Raptor. *n* = 3 per group. (**E**, **F**) Representative electron microscopy images showing the number of autophagosomes in cells (red arrow indicates an autophagosome fusing with a lysosome) and quantitative analysis of autophagosome numbers. *n* = 9 images per group. (**G**, **H**) Western blot analysis and quantitative analysis of total tau protein and phosphorylated tau protein levels in HEK293-P301S cells 36 h after transfection with Raptor. *n* = 3 per group. Unpaired t-tests were used for data analysis in all panels. All data were presented as mean ± SEM. ns indicates no statistically significant difference, *p < 0.05, **p < 0.01, ***p < 0.001, ****p < 0.0001

To investigate the impact of Raptor overexpression on autophagy and tau degradation, HEK293T cells were transfected with an eGFP-P301S plasmid (HEK293T-P301S) to express human P301S mutant tau tagged with eGFP. Raptor plasmid was then co-transfected into both HEK293T and HEK293T-P301S cells. Western blot analysis revealed that Raptor overexpression activated mTORC1 and impaired autophagy in HEK293T cells and HEK293T-P301S cells, as evidenced by increased mTOR levels, an elevated p-P70 S6K/P70S6K ratio, a decreased LC3-II/LC3-I ratio, and accumulation of P62 (Fig. 5A-D). Electron microscopy results indicated that Raptor overexpression inhibited the number of autophagosomes in HEK293T-P301S cells (Fig. 5E, F). Unexpectedly, Raptor overexpression did not alter total tau levels or its phosphorylation at Ser404, Ser396, and Thr205 (Fig. 5G, H).

### Raptor knockdown reduced tau protein levels through the activation of autophagy in vitro

**Fig. 6:**
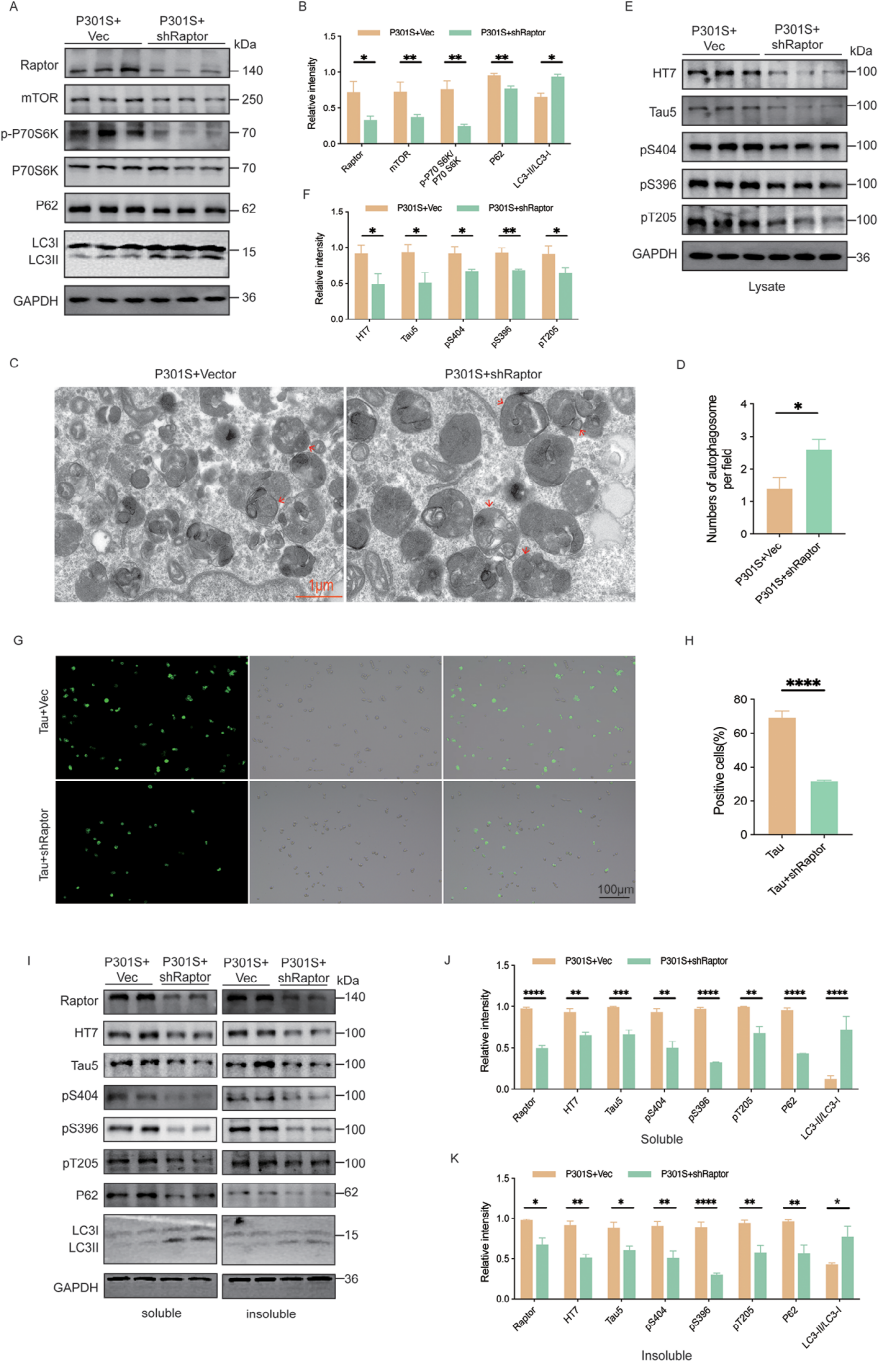
Raptor knockdown reduced tau protein levels through the activation of autophagy in vitro. (**A**, **B**) Western blot analysis and quantitative analysis of Raptor, mTOR, p-P70 S6K, P70 S6K, P62, and LC3 expression in HEK293T/P301S cells 24 h after transfection with shRaptor. *n* = 3 per group. (**C**, **D**) Representative electron microscopy images showing the number of autophagosomes in cells (red arrow indicates an autophagosome fusing with a lysosome) and quantitative analysis of autophagosome numbers. *n* = 9 images per group. (**E**, **F**) Western blot analysis and quantitative analysis of total tau protein and phosphorylated tau protein levels in HEK293T/P301S cells 24 h after transfection with shRaptor. *n* = 3 per group. (**G**, **H**) Representative fluorescence microscopy images of tau protein expression in HEK293T-Tau cells 24 h after transfection with shRaptor, along with the corresponding quantitative analysis. *n* = 5 per group. (**I**) Western blot analysis of Raptor, total tau protein, phosphorylated tau protein, P62, and LC3 in sarkosyl-soluble and sarkosyl-insoluble protein fractions extracted from HEK293T-P301S cells. *n* = 3 per group. (**J**) Quantification of protein levels in the sarkosyl-soluble fraction. (**K**) Quantification of protein levels in the sarkosyl-insoluble fraction. Unpaired t-tests were used for data analysis in all panels. All data were presented as mean ± SEM. ns indicates no statistically significant difference, *p < 0.05, **p < 0.01, ***p < 0.001, ****p < 0.0001

To further elucidate the role of Raptor knockdown in tau degradation, we co-transfected HEK293T-P301S cells with shRaptor plasmid and examined the expression of autophagy-related proteins. Raptor knockdown decreased the levels of mTOR and p-P70 S6K, while increasing the LC3-II/LC3-I ratio and reducing P62 levels (Fig. 6A, B). Electron microscopy results demonstrated that Raptor knockdown increased the number of autophagosomes in HEK293T-P301S cells (Fig. 6C, D). These findings indicated that Raptor knockdown activated autophagy through the inhibition of mTORC1 signaling.

Raptor knockdown also reduced total tau levels and decreased phosphorylation at Ser404, Ser396, and Thr205 (Fig. 6E, F). These findings were corroborated in HEK293T cells stably expressing GFP-Tau (HEK293T-Tau cells), where Raptor knockdown similarly reduced tau levels as assessed by cellular fluorescence imaging (Fig. 6G, H).

As abnormal forms of tau are highly insoluble in nonionic and ionic detergents [32, 33], we isolated the Sarkosyl-soluble and -insoluble fractions to investigate the effect of Raptor knockdown. Similar results were observed in both soluble and insoluble fractions of Sarkosyl, further supporting the role of Raptor knockdown in promoting autophagy and tau degradation (Fig. 6I-K).

### USP9X is upregulated in P301S mice

**Fig. 7:**
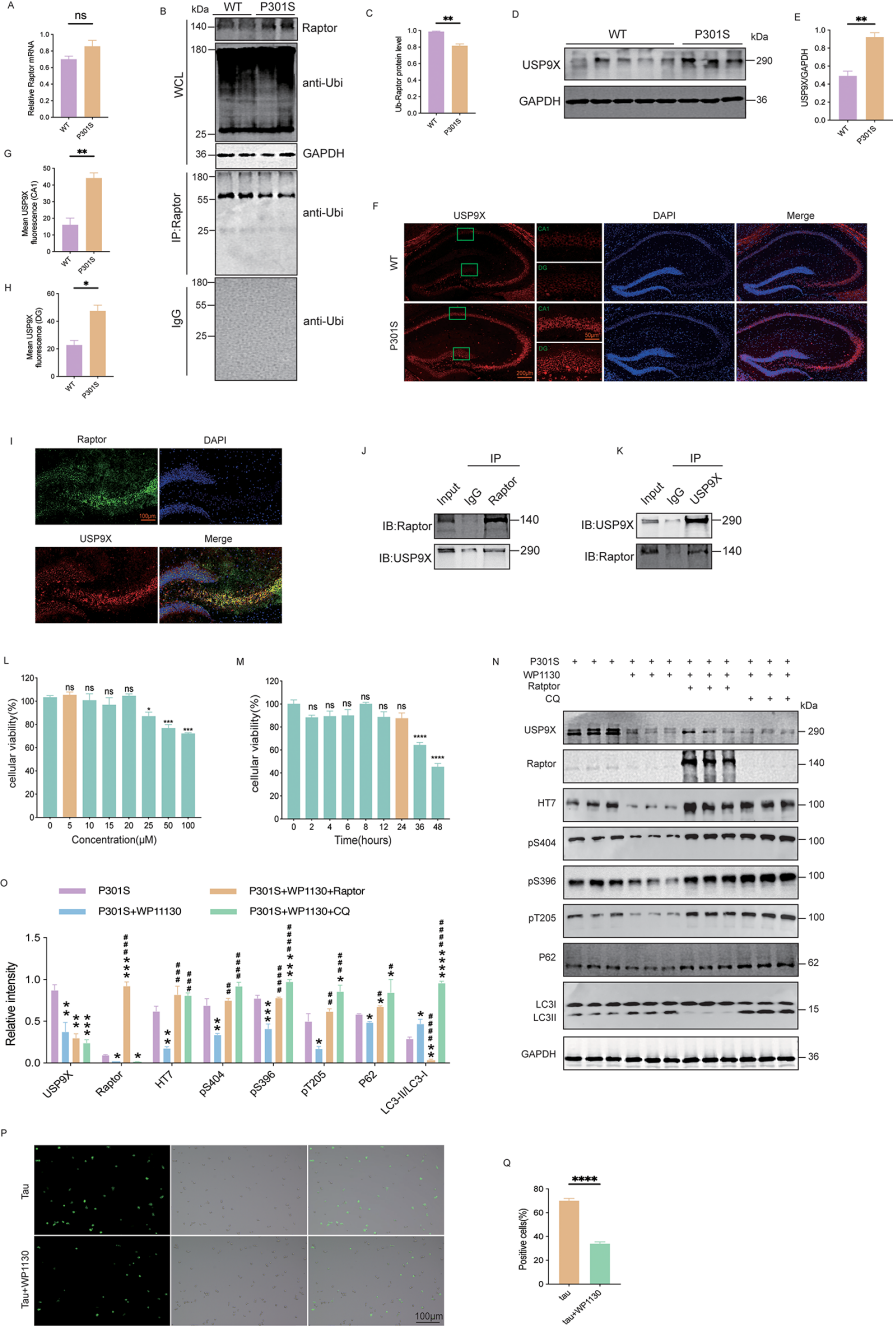
USP9X was upregulated in P301S mice and deubiquitinated Raptor. (**A**) The levels of Raptor mRNA showed no significant changes in the hippocampus of 6-month-old P301S mice compared to WT mice. *n* = 3 mice per group. (**B**, **C**) Raptor ubiquitination levels were compared in the hippocampus of 6-month-old P301S mice and WT mice, along with quantitative analysis. (**D**, **E**) Western blot analysis and quantitative analysis of USP9X levels in the hippocampus of 6-month-old P301S mice compared to WT mice. *N* ≥ 3 per group. (**F**-**H**) Immunofluorescence staining of USP9X (red) in the hippocampus of 6-month-old WT and P301S mice. Nuclei were counterstained with DAPI (blue). Quantitative analysis of USP9X fluorescence intensity is also shown. *n* = 3 mice per group. (**I**) Immunofluorescence staining showing co-localization of Raptor (green) and USP9X (red) in the CA3 and dentate gyrus (DG) regions of the hippocampus in P301S mice. Nuclei were counterstained with DAPI (blue). (**J**, **K**) Co-immunoprecipitation experiment demonstrating the interaction between USP9X and Raptor in the hippocampus of P301S mice. (**L**) HEK293T cells were treated with increasing concentrations of WP1130 in serum-free medium for 24 h. Cell viability was assessed using the CCK-8 assay by measuring absorbance at 450 nm (*n* = 6 replicates per concentration). (**M**) HEK293T cells were treated with 5 µM WP1130, and cell viability was measured at various time points using the CCK-8 assay (*n* = 6 replicates per time point). (**N**, **O**) Western blot analysis of USP9X, Raptor, total tau, phosphorylated tau, P62, and LC3 levels in HEK293T-P301S cells treated with 5 µM WP1130 for 24 h. The effects of WP1130 were reversed by overexpression of Raptor or treatment with CQ. *n* = 3 per group. *p < 0.05, **p < 0.01, ***p < 0.001, ****p < 0.0001 vs. P301S group; ^#^*p* < 0.05, ^##^*p* < 0.01, ^###^*p* < 0.001, ^####^*p* < 0.0001 vs. P301S + WP1130 group. (**P**, **Q**) Representative fluorescence microscopy images showing the effect of 5 µM WP1130 treatment for 24 h on tau protein expression in HEK293T-Tau cells along with the corresponding quantitative analysis. *n* = 5 per group. Unpaired t-tests were used for data analysis in (**A**, **C**, **E**, **G**, **H**, **Q**). One-way ANOVA followed by Tukey’s multiple comparisons test was used for data analysis in (**L**, **M**, **O**). All data were presented as mean ± SEM.*p < 0.05, **p < 0.01, ***p < 0.001, ****p < 0.0001

To investigate the mechanisms underlying the increased Raptor protein levels in the hippocampus of P301S mice, we quantified Raptor mRNA levels using qPCR. Although Raptor mRNA levels were slightly elevated in the hippocampus of 6-month-old P301S mice compared to WT mice, the difference was not statistically significant (Fig. 7A). We hypothesized that post-translational modifications might contribute to the increase in Raptor protein levels, as previous studies have reported that Raptor is subject to ubiquitination-mediated regulation [34–36]. Consistent with our hypothesis, we found decreased ubiquitination levels of Raptor in 6-month-old P301S mice compared to WT mice (Fig. 7B, C).

A study showed that the deubiquitinating enzyme USP9X interacted with Raptor in HEK-293T cells and was associated with mTORC1 signaling [34]. Using western blot and immunofluorescence, we detected increased USP9X levels in the hippocampus of 6-month-old P301S mice compared to WT mice (Fig. 7D-H). Based on these findings, we hypothesize that elevated USP9X levels contribute to increased Raptor levels, leading to mTORC1 activation and subsequent impairment of autophagy in P301S mice.

### Raptor is deubiquitinated by USP9X

To further substantiate the interaction between USP9X and Raptor, we conducted immunofluorescence staining in mouse cortex and hippocampal tissue, revealing co-localization of both proteins (Fig. S4A; Fig. 7I). Furthermore, co-immunoprecipitation experiments using mouse hippocampal lysates confirmed an interaction between endogenous USP9X and Raptor, providing evidence that supports their potential in vivo association (Fig. 7J, K). This finding was consistent with previous studies that demonstrated the interaction between USP9X and Raptor in HEK293T cells [34, 37].

To investigate the functional consequences of USP9X inhibition, we used WP1130, a deubiquitinating enzyme inhibitor known to directly inhibit USP9X activity, which leads to increased Raptor ubiquitination levels and reduces Raptor levels within hours [34, 37]. We first conducted a half-life experiment and found that WP1130 promoted the degradation of Raptor following cycloheximide (CHX) treatment (Fig. S4B, C). Autophagy and the proteasome system are two key protein degradation pathways in cells. To determine the potential degradation pathways, we employed the proteasome inhibitor MG132 and the lysosomal inhibitor chloroquine (CQ). Western blot analysis revealed that MG132 treatment blocked WP1130-induced downregulation of RAPTOR, while chloroquine treatment had no effect (Fig. S4D, E).

To determine the optimal treatment conditions in HEK293T-P301S cells, we performed a concentration and time-course experiment using CCK-8 assays. Cell viability significantly decreased at concentrations exceeding 25 µM for 24 h and after 36 h of treatment with 5 µM WP1130 (Fig. 7L, M). Based on these results, we treated HEK293T-P301S cells with 5 µM WP1130 for 24 h for subsequent analyses. Western blot analysis revealed that WP1130 treatment effectively reduced USP9X protein levels, accompanied by a decrease in Raptor levels, an increase in the LC3-II/LC3-I ratio, and a decrease in P62 levels (Fig. 7N, O). These findings indicated that USP9X inhibition promoted autophagy in HEK293T-P301S cells. Furthermore, WP1130 treatment significantly reduced both total tau and phosphorylated tau levels at Ser396, Ser404, and Thr205, as demonstrated by western blot and immunofluorescence analyses (Fig. 7N-Q).

To explore whether the promotion of autophagy and tau degradation by WP1130 could be reversed, we overexpressed Raptor or treated the cells with the autophagy inhibitor CQ. Our previous study showed that treating HEK293T-P301S cells with 20 µM CQ for 4 h significantly increased P62 and LC3B-II levels, thereby blocking autophagosome-lysosome fusion, and increased total tau and phosphorylated tau levels [38]. Compared to WP1130 treatment alone, WP1130 treatment combined with Raptor overexpression or CQ treatment reversed the autophagy-promoting effect of WP1130, leading to increased total tau and phosphorylated tau levels at Ser404, Ser396, and Thr205 (Fig. 7N, O). These results indicated that the promotion of autophagy and tau degradation by WP1130 could be reversed by overexpressing Raptor or inhibiting autophagy with CQ.

In conclusion, our findings suggested that elevated USP9X levels in the hippocampus of P301S mice may contribute to increased Raptor protein levels. Pharmacological inhibition of USP9X with WP1130 promoted autophagy and reduced tau accumulation in vitro, which can be reversed by overexpressing Raptor or CQ.

## Discussion

Our study revealed an intriguing connection between mTORC1 activity, Raptor levels, and tau pathology in P301S mice, a model of tauopathy. We observed elevated ratios of p-mTOR/mTOR and p-P70 S6K/P70 S6K, decreased LC3-II/LC3-I ratios, and increased P62 levels in P301S mice, along with a reduction in autophagosomes observed through electron microscopy. These findings indicated mTORC1 hyperactivation and impaired autophagosome formation. Our results were consistent with previous studies demonstrating a link between tau accumulation and mTORC1 activation via the TIA1-amino acid-mTORC1 signaling pathway [13, 39]. While mTORC1 and its inhibition by rapamycin have been extensively studied in AD models [40], the role of Raptor, a key mTORC1 component, in neurodegenerative diseases remains largely unexplored. Previous reports have shown elevated Raptor and phospho-Raptor levels in post-mortem hippocampal tissues of late-stage AD patients [10]. Our findings demonstrated increased Raptor levels in the hippocampus of 6- and 9-month-old P301S mice, but not in 3-month-old mice (Fig S1D, E). Notably, immunofluorescence revealed prominent Raptor signals in the CA3 region of P301S mice. Therefore, we targeted Raptor knockdown specifically in the CA3 region of 6-month-old P301S mice. Raptor knockdown significantly attenuated mTORC1 signaling, enhanced autophagosome formation, and reduced tau accumulation in P301S mice. This reduction in tau pathology was accompanied by improvements in synaptic integrity, reduced neuronal loss, and enhanced learning and memory performance.

To further investigate the influence of Raptor on tau protein at the cellular level, we compared Raptor protein levels in HEK293T cells and HEK293T-P301S cells. Surprisingly, we observed no significant difference (results not displayed), possibly due to the non-neuronal origin of HEK293T cells. However, leveraging the ease of transfection in this cell line, we proceeded to overexpress and knock down Raptor in HEK293-P301S cells. As expected, Raptor overexpression activated mTORC1 and suppressed autophagosome formation, while Raptor knockdown elicited the opposite effects. Interestingly, while Raptor overexpression did not alter tau levels, Raptor knockdown significantly reduced total tau, phosphorylated tau, and both soluble and insoluble tau fractions.

To elucidate the mechanism underlying elevated Raptor protein levels in P301S mice, we first examined Raptor mRNA expression. No significant difference in Raptor mRNA levels was observed between P301S and wild-type mice, suggesting that transcriptional regulation is unlikely the primary cause. We then investigated the possibility of impaired Raptor degradation in P301S mice. Indeed, we found reduced Raptor ubiquitination in the hippocampus of 6-month-old P301S mice compared to WT mice. This finding suggested that impaired Raptor degradation may contribute to the elevated Raptor levels in P301S mice. Previous studies have shown that Raptor is regulated by deubiquitinases such as USP9X [34], OTUB1 [36], and UCH-L1 [35]. Given that OTUB1 can modulate mTORC1 signaling and autophagy by regulating Raptor and Deptor [41, 42], it is plausible to hypothesize an OTUB1-Raptor-mTORC1 axis contributing to autophagy impairment and tau upregulation. However, there is currently no evidence indicating increased OTUB1 expression in the P301S mouse model. Furthermore, a recent study demonstrated that OTUB1 expression is gradually downregulated with increasing tau-seeded tau aggregation, suggesting a potential protective mechanism [43]. We also examined the expression of UCH-L1 in the hippocampus of 6-month-old P301S mice and found no significant differences compared to the WT group (results not shown). Previous studies have reported a positive correlation between USP9X and MAPT in zebrafish and DU145 cells [25]. However, whether a similar relationship exists in P301S mice remains unknown. Since USP9X is located on the X chromosome, we focused on 6-month-old male P301S and WT mice for USP9X analysis. Remarkably, we observed significantly elevated USP9X expression in the hippocampus of P301S mice, with clear colocalization of USP9X and Raptor. Based on these findings, we proposed that elevated USP9X in P301S mice led to Raptor accumulation, subsequent mTORC1 activation, impaired autophagy, and ultimately, increased tau accumulation and phosphorylation.

The relationship between USP9X, mTORC1 signaling, and autophagy remains complex and incompletely understood. We compiled a table summarizing studies on USP9X and autophagy, including the cell types, treatment (e.g., drug concentration and duration, si-USP9X/sh-USP9X), results (e.g., autophagy promotion/inhibition or changes in autophagy markers), and relevant references (Table S1). In our study, we observed that autophagy was promoted in HEK293T-P301S cells treated with 5 µM WP1130 for 24 h. We also tested 5 µM WP1130 for 2 h, but no significant changes in autophagy levels were observed (data not shown). Additionally, we found that autophagy was promoted in n2a-app cells treated with 5 µM WP1130 for 24 h (Figs. S6A, B), while the same treatment in SH-SY5Y cells resulted in significant cell death. Based on the references in the table and our results, it can be concluded that USP9X regulates multiple points in the autophagy pathway, including mTOR, Beclin1, and P62. However, the influence of USP9X on autophagy appeared to be related to the concentration and duration of WP1130 administration, as well as the specific cell type used. It is crucial to acknowledge that WP1130 exhibits non-specific inhibition, also targeting the DUB activity of USP5, USP14, and UCH37 [44]. It is also important to consider the findings of Stefan Drießen, who noted that ULK1 levels in the soluble fraction remained unaltered in cells deficient in USP9X or in which USP9X was knocked down by siRNA. Furthermore, LC3 turnover occurred normally in USP9X-negative HCT116 cells but was blocked by WP1130 treatment in both WT and USP9X-negative cells. These observations suggested that additional DUBs or a combination of DUBs may mediate the WP1130 effect on ULK1 [44].

The effect of WP1130 on USP9X protein levels has been a subject of debate within the literature. While some studies indicated that WP1130 solely inhibited USP9X activity without affecting protein levels [34], others demonstrated a reduction in USP9X protein expression following WP1130 treatment [45]. Our findings aligned with the latter, showing a decrease in USP9X protein levels in HEK293T-P301S cells treated with 5 µM WP1130 for 24 h. We also observed a significant decrease in Raptor levels following WP1130 treatment, consistent with previous research [34].

Interestingly, WP1130 treatment activated autophagy, accompanied by reduced levels of both total tau and phosphorylated tau in HEK293T-P301S cells. Furthermore, overexpression of Raptor or treatment with CQ reversed the WP1130-mediated increase in autophagy and decrease in tau protein, providing further evidence that USP9X inhibition influences tau degradation via a Raptor- and autophagy-dependent mechanism. Additionally, we found through co-immunoprecipitation that USP9X interacted with tau in the hippocampus of P301S mice and in HEK293T-Tau cells (Fig. S6A-D). Whether USP9X directly maintains tau protein stability through deubiquitination also warrants further investigation. Therefore, targeting Raptor and USP9X holds promise as a therapeutic strategy for Alzheimer’s disease.

The study also has certain limitations. While HEK-293T cell lines supported our in vivo findings, further research should be conducted in neuronal cell types to validate our results. In addition, the cortex of P301S mice also showed a significant reduction in Raptor. Future studies should consider applying WP1130 in mice to investigate tau pathology in the cortex. Additionally, using siRNA targeting USP9X is necessary to avoid potential interference from WP1130 at other sites. Relying solely on fluorescence co-localization and co-immunoprecipitation (co-IP) is insufficient to demonstrate a direct co-localization between Raptor and USP9X; future studies employing proximity ligation assays (PLA) or fluorescence resonance energy transfer (FRET) will provide more direct evidence of their interaction. We focused on male mice to minimize variability introduced by hormonal fluctuations associated with the estrous cycle in females. This decision allowed us to isolate the specific role of USP9X in tauopathy without the confounding factor of sex hormones. However, we recognize that our findings cannot be directly extrapolated to females, and future studies incorporating both sexes are essential to determine if the observed effects are sex-dependent. Furthermore, male mice also express USP9Y, and there is evidence suggesting that USP9Y, along with seven other genes, is among the commonly differentially expressed genes in the dorsolateral prefrontal cortex of subjects with AD or major depressive disorder [46]. Additionally, USP9Y displays significant gender-linked expression in the adult brain and shows diminished expression in Alzheimer’s disease. In DU145 cells, knockdown of USP9X/Y leads to a reduction in MAPT expression levels [25]. We are currently planning follow-up studies that will include female mice and investigate the role of USP9Y in male mice.

## Conclusion

1. Raptor protein levels are elevated in the hippocampus of P301S mice at 6 and 9 months of age. Knockdown of Raptor in the CA3 region of the hippocampus in 6-month-old P301S mice improves autophagy, reduces the accumulation of tau protein and phosphorylated tau protein, ameliorates synapse and neuron loss, and enhances learning and memory.
2. Knockdown of Raptor in HEK293T-P301S cells promotes autophagosome formation and tau degradation.
3. The elevated levels of USP9X in the hippocampus of 6-month-old P301S mice may contribute to the increased Raptor expression.
4. Treatment with the USP9X inhibitor WP1130 enhances autophagy and tau degradation in HEK293T-P301S cells, which can be reversed by Raptor overexpression or treatment with CQ.

## Materials and methods

See Table 1.

**Table 1:** Antibodies used in this study

Antibody	Host	Dilution WB	Dilution IHC	Dilution IF	Source
Anti-Raptor	Rabbit	1:1000			2280, Cell Signaling Technology
Anti-Raptor	Rabbit			1:100	20984-1-AP, Proteintech
HT7	Mouse	1:1000			MN1000, Thermo Fisher Scientific
Tau5	Mouse	1:1000			ab80579, Abcam
Anti-tau(pS396)	Rabbit	1:1000	1:200		11,102, Signalway Antibody
Anti-tau(pS404)	Rabbit	1:1000	1:200		11,112, Signalway Antibody
Anti-tau(pT205)	Rabbit	1:1000			11,108, Signalway Antibody
Anti-GAPDH	Mouse	1:1000			60004-1-Ig, Proteintech
Anti-p-mTOR(Ser2448)	Rabbit	1:500			R25033, Zenbio
Anti-mTOR	Mouse	1:5000			66888-1-Ig, Proteintech
Anti-p-P70 S6K(Thr421/Ser424)	Rabbit	1:500			380,880, Zenbio
Anti-P70 S6K	Rabbit	1:1000			14485-1-AP, Proteintech
Anti-LC3B	Rabbit	1:1000			ab51520, Abcam
Anti-p62 /SQSTM1	Rabbit	1:1000			18420-1-AP, Proteintech
Anti-SYN	Rabbit	1:1000			17785-1-AP, Proteintech
Anti-PSD95	Rabbit	1:1000			A0131, Abclonal
Anti-ubiquitin	Mouse	1:1000			sc-8017, Santa Cruz Biotechnology
Anti-USP9X	Rabbit	1:1000		1:100	55054-1-AP, Proteintech
Anti-NeuN	Rabbit			1:100	66836-1-Ig, Proteintech
Anti-Iba-1	Rabbit	1:1000			10904-1-AP, Proteintech
Anti-Iba-1	Rabbit	1:1000		1:500	019-19741, Wako
Anti-GFAP	Rabbit	1:1000			60190-1-Ig, Proteintech
Anti-GFAP	Mouse			1:400	3670 S, Cell Signaling Technology
Anti-EGFP	Mouse			1:2000	Gb12602, Servicebio
Anti-mouse IgG	Goat	1:10000			SA00001-1, Proteintech
Anti-rabbit IgG	Goat	1:10000			SA00001-2, Proteintech
Anti-mouse IgG	Donkey			1:100	GB21401, Servicebio
Anti-rabbit IgG	Goat			1:100	GB21303, Servicebio
Anti-rabbit IgG	Goat		1:200		G1212, Servicebio
Anti-mouse IgG	Goat	1:2000			M21004, Abmart

### Animals

P301S transgenic mice [B6;C3-Tg(Prnp-MAPT/P301S)PS19Vle/J] and their wild-type littermates were obtained from the Jackson Laboratory (Bar Harbor, ME, USA). The animals were housed in a specific pathogen-free facility under a 12-hour light/dark cycle with ad libitum access to food and water. Mice were randomly allocated into cages, with 4–5 animals per cage. All animal experiments were conducted in compliance with the “Policies on the Use of Animals and Humans in Neuroscience Research” revised and approved by the Society for Neuroscience in 1995, the Guidelines for the Care and Use of Laboratory Animals of the Ministry of Science and Technology of the People’s Republic of China, and the Institutional Animal Care and Use Committee at Tongji Medical College, Huazhong University of Science and Technology. The study protocol was reviewed and approved by the Ethics Committee of Tongji Medical College, Huazhong University of Science and Technology (Wuhan, China).

### Cell culture, transfection and plasmids

HEK293T cells or HEK293T-Tau cells were maintained in Dulbecco’s Modified Eagle Medium (DMEM) supplemented with 10% fetal bovine serum (FBS) and cultured at 37 °C in a humidified atmosphere containing 5% CO2. For transfection experiments, HEK293T cells or HEK293T-Tau were seeded into 12-well plates at a density that allowed them to reach 80% confluence within 24 h. Prior to transfection, the culture medium was replaced with fresh complete DMEM. Transfection complexes were prepared by mixing 1 µg of plasmid DNA, 1 µL of transfection reagent (TF201201, Neofect, Beijing, China), and 100 µL of serum-free Opti-MEM reduced serum medium (Invitrogen, Carlsbad, CA, USA) according to the manufacturer’s instructions. The complexes were incubated at room temperature for 30 min to allow for the formation of lipid-DNA complexes. The transfection mixture was then added dropwise to the cells and incubated for 24–36 h at 37 °C. Transfection efficiency was assessed using fluorescence microscopy based on the expression of the plasmid-encoded fluorescent reporter protein.

The pLKO.1-shRNA-Raptor plasmids (#1857) were obtained from Addgene (USA). EGFP-pRK5-HA-Raptor was obtained from Tsingke Biotechnology (Beijing, China). EGFP-C1-P301S was obtained from OBIO Biotech (Shanghai, China).

### Drug administration

WP1130 (HY-13264, MedChemExpress) was dissolved in dimethyl sulfoxide (DMSO) to prepare a 5 mM stock solution, which was aliquoted and stored at − 80 °C, protected from light. For cell treatment, the stock solution was further diluted with the appropriate cell culture medium to achieve a final working concentration of 5 µM. The final DMSO concentration in the treated cells was maintained at or below 0.1% (v/v) to minimize any potential vehicle-related effects on cell viability or cellular processes.

CQ (C6628, Sigma-Aldrich) and MG132 (HY-13259, MedChemExpress) were prepared as 5 mM stock solutions in DMSO, with a final working concentration of 20 µM. CHX (S7418, Selleck) was also prepared in DMSO, with a working concentration of 20 µg/ml.

### Stereotactic brain injection

pAAV-U6-shRNA(Rptor)-CMV-EGFP-WPRE and corresponding control vector pAAV-U6-shRNA(NC2)-CMV-EGFP-WPRE were generated by OBIO Biotech (Shanghai, China). After being fixed on stereotaxic apparatus with adequate anesthesia, mice were injected with 500 nl of virus into the hippocampal CA3 area bilaterally (AP = -2.0 mm, ML = ± 2.5 mm, DV = -2.3 mm) [47]. Injection rate was maintained at 100 nl/min, and the needle syringe was kept in situ for an additional 10 min after the virus was fully injected. Following the injection, mice were placed on a heating pad to maintain body temperature until they recovered from anesthesia and were then returned to their home cages.

### Behavior tests

One month following the stereotactic injection, behavioral experiments were performed to assess the spatial learning and memory abilities of the mice. The NOR test was conducted in a 50 cm × 50 cm arena. Twenty-four hours prior to the test, mice were habituated to the empty arena for 5 min. On the test day, during the familiarization phase, mice were placed in the arena containing two identical objects (A and A′) located at opposite ends of the arena and allowed to explore for 5 min. After a 1-hour inter-trial interval, one of the familiar objects (A′) was replaced with a novel object (B), and the mice were returned to the arena for the test phase, during which they were allowed to explore both objects for 5 min. The behavior of the mice was recorded using an overhead video camera. The time spent exploring each object during the test phase was measured, with exploration defined as directing the nose toward the object at a distance of less than 2 cm and/or touching the object with the nose. The recognition index was calculated as the ratio of time spent exploring the novel object (TB) to the total exploration time (TA + TB), i.e., TB/ (TA + TB).

The Morris water maze (MWM) test was used to assess the learning and memory abilities of laboratory animals in the context of spatial position and orientation. It was performed as follows: during the spatial learning phase, mice underwent training to locate a hidden platform positioned consistently below the water surface over a period of five consecutive days. Training sessions were conducted between 12:00 pm and 17:00 pm. In each training session, mice were gently placed into the water maze from one of the three remaining quadrants (without the target platform), with their orientation facing the pool wall. If the platform was not located within 60 s, mice were guided to the platform and allowed to remain on it for an additional 30 s. On the seventh day, the platform was removed, and mice were placed in the water maze for 60 s to assess spatial memory retention. The movement trajectories of the mice were recorded and subsequently analyzed using the MWZ-100 system developed by Techman (Techman, China).

### Immunohistochemical

Mouse brain slices (5 μm thick, paraffin-embedded) were baked at 55 °C for 2 h and then immersed in xylene for an additional 2 h to ensure complete deparaffinization. The slices were then rehydrated through a graded ethanol series (100%, 100%, 95%, 85%, and 75%) for 5 min each. To enhance antigen retrieval, the brain slices were immersed in citric acid buffer (10 mM, pH 6.0) and heated in a microwave for 15 min. Subsequently, the slices were incubated with 3% H2O2 for 30 min to quench endogenous peroxidase activity and then blocked in a 5% BSA solution containing 0.5% Triton X-100 for 30 min to minimize non-specific antibody binding. The slices were then incubated with the primary antibody at 4 °C for 24–48 h, followed by incubation with the secondary antibody at 37 °C for 1 h. Immunoreactivity was visualized using a DAB reagent (G1212, Servicebio) according to the manufacturer’s instructions. Finally, the slices were dehydrated through a graded ethanol series (75%, 85%, 95%, 100%, and 100%) for 5 min each, cleared in xylene for 5 min, and mounted with neutral balsam for long-term preservation and microscopic analysis.

### Immunofluorescence staining

Following deparaffinization, dehydration, and antigen retrieval, mouse brain slices were washed with PBS (3 × 5 min) and blocked in 5% donkey serum with 0.5% Triton X-100 for 30 min. Slices were then incubated with primary antibodies (Table 1) at 4 °C for 24–48 h, followed by secondary antibody incubation at 37 °C for 1 h. After washing with PBS (3 × 5 min), slices were stained with DAPI (G1012, Servicebio) for 5 min at room temperature, sealed with an anti-fluorescence quencher (G1401, Servicebio), and imaged using an scanning microscope (OLYMPUS SV120).

For the homologous dual-label triple-color fluorescence staining, we used a three-color multiple fluorescence staining kit (RC0086-23R, RecordBio). Paraffin sections were deparaffinized, subjected to antigen retrieval, and incubated with 3% H2O2 for 15 min. After blocking with 5% BSA solution containing 0.5% Triton X-100 for 30 min, slices were incubated with primary antibodies at 4 °C for 24 h. HRP-conjugated secondary antibodies were applied for 50 min at room temperature, followed by TYR-520 fluorescent dye incubation for 15 min. Antibodies were stripped by incubating the slices in antigen retrieval solution at 95 °C for 30 min. The process was repeated from the 3% H2O2 incubation step, using TYR-570 fluorescent dye. DAPI was added for 10 min at room temperature. Slices were sealed with an anti-fluorescence quencher and imaged using an scanning microscope (OLYMPUS SV120).

### Nissl’s staining

After deparaffinization and gradient alcohol dehydration, slices were washed with PBS three times for 5 min each. Slices were stained with 0.5% toluidine blue reagent (G1036, Servicebio) for 2–5 min. If the slices were overstained, differentiation was performed using 0.1% glacial acetic acid. The stained slices were then baked and mounted with neutral balsam. Imaging was conducted using an scanning microscope (OLYMPUS SV120).

### TUNEL staining

The One-step TUNEL In Situ Apoptosis Kit (Elabscience) was used for detecting apoptotic cells in brain slices. After deparaffinization, rehydration, antigen retrieval, and blocking, the slices were incubated with proteinase K working solution at 37 °C for 30 min. The permeabilized samples were then washed three times with PBS for 5 min each. TdT Equilibration Buffer was added, and the slices were equilibrated at 37 °C in a humidified chamber for 20 min. The labeling solution was applied, and the slices were incubated in a humidified chamber at 37 °C for 60 min in the dark. The slices were washed three times with PBS for 5 min each and stained with DAPI reagent for 5 min at room temperature. Finally, the slices were sealed with an anti-fluorescence quencher and imaged using an scanning microscope (OLYMPUS SV120).

### Golgi staining

The FD Rapid GolgiStainTM Kit (FD Neuro Technologies, PK401) was used for Golgi staining. Mice were deeply anesthetized, and their brains were removed and immersed in a mixture of solution A and B (1:1) for 3 weeks. The brains were then transferred to solution C for 7 days. Following this, the brains were placed on an oscillating tissue slicer and cut into 100 μm thick slices. The slices were air-dried in the dark and then stained with a mixture of solution D, E, and double distilled water (1:1:2) according to the manufacturer’s instructions. Images were acquired using an optical microscope (Nikon, Japan).

### Co-immunoprecipitation

Hippocampal regions were isolated and mechanically homogenized in lysis buffer (Biosharp) containing protease inhibitor cocktail, PMSF, and phosphatase inhibitors A and B. The homogenate was then centrifuged at 12,000 rpm for 20 min at 4 °C. The supernatant was diluted to a concentration of 4.8 µg/µL. For immunoprecipitation, 2 mg of protein sample was incubated with 4 µg of primary antibody overnight at 4 °C with gentle rotation. Following this, 50 µL of Protein A + G Agarose (P2012, Beyotime) was added to the sample and incubated for 4 h. The agarose beads were then washed three times. Proteins bound to the agarose were resuspended in buffer containing 50 mM Tris-HCl (pH 6.8), 2% SDS, and 10% glycerol, and boiled for 10 min. The collected protein sample was then analyzed by Western blot.

### qPCR

Total RNA was extracted from the hippocampal region of virus-infected mice using Trizol reagent (Thermo Fisher Scientific, 15596018). Complementary DNA (cDNA) was synthesized using the Transcription Reagent Kit (Takara, RR037). Quantitative PCR (qPCR) was performed using the One-Step SYBR PrimeScript PLUS RT-PCR Kit (Takara, RR096A) according to the manufacturer’s instructions. The reaction mixture consisted of 1 µl each of forward and reverse primers, 1 µl of cDNA, 3 µl of diethylpyrocarbonate-treated water (DEPC H2O), and 5 µl of SYBR Green PCR master mix. Real-time PCR was carried out and analyzed using an ABI StepOnePlus Real-Time PCR System (Applied Biosystems). Primers for Raptor were: F 5′-GGTGGAACTGAGGTTACATGATG-3′ and R 5′-TCCTGTTTGTGTGCAACTGCT-3′.

### Protein extraction and Western blot

To lyse cells or tissues, RIPA buffer was prepared with the appropriate protease and phosphatase inhibitors (G2006, Servicebio, Wuhan) and kept on ice. The lysate was subjected to sonication several times, and the protein concentration was measured using the BCA assay (Beyotime Biotechnology, Shanghai, China). The samples were then transferred to 1.5 ml Eppendorf tubes and mixed with loading buffer. Complete denaturation of the samples occured by placing them in a metal bath at 95 °C for 10 min.

To prepare sarkosyl soluble and insoluble fractions, the sample was mechanically homogenized in 10 volumes of pre-cooled lysis buffer (10 mM Tris-HCl, pH 7.4, 0.8 M NaCl, 1 mM EGTA, 10% sucrose) and then centrifuged at 20,000 ×g for 20 min at 4 °C. The supernatant (S1) was transferred to a new Eppendorf tube, and the pellet was re-homogenized in 5 volumes of lysis buffer and centrifuged again at 20,000 ×g for 20 min. The second supernatant (S2) was combined with S1 and incubated with 1% N-lauroylsarkosynate for 1 h at room temperature while shaking. The sample was then centrifuged at 100,000 ×g for 1 h at 4 °C. The supernatant was transferred to a new Eppendorf tube and designated as the soluble fraction. The pellet was re-suspended in 50 mM Tris–HCl (pH 7.4) and stored as the sarkosyl insoluble fraction.

Protein samples were separated by SDS-PAGE and transferred to a nitrocellulose filter membrane (10600002, Whatman). The membrane was then blocked with a rapid blocking solution (PS108, epizyme) for 15 min or 5% BSA for 1 h. Following blocking, the membrane was incubated with primary antibodies overnight at 4 °C and then with the corresponding secondary antibodies for 1 h. The antibodies used in this study are listed in Table 1. Protein bands were visualized using an ECL Imaging System (610007-8Q, Clinx Science Instruments Co., Ltd.). If necessary, the nitrocellulose filter membrane was stripped using a rapid stripping buffer (CW0056S, cwbio) for 15 min and then re-blocked before incubating with another primary antibody. Quantitative analysis of the blots was performed using ImageJ software (Fiji).

### Cell viability assays (CCK-8 assays)

HEK293 cells were seeded into a 96-well plate at a density of 10,000 cells per well, with a blank control group included. Groups of six replicates were treated with drugs at different concentrations or for different durations. Following drug treatment, 10 µL of CCK-8 reagent (Dojindo, Japan) was added to each well, and the absorbance at 450 nm was measured every 30 min until most of the OD values approached 1.0. Cell viability was then calculated based on the absorbance values.

### Transmission electron microscopy

The numbers of autophagosomes and synapses were quantitatively analyzed using electron microscopy. Briefly, the brains were fixed with 2.5% glutaraldehyde and then cut into 50 μm thick slices. The entire CA3 region of the hippocampus was selected and post-fixed in 1% osmium tetroxide (dissolved in 0.1 M PB) for 25 min, followed by staining with 1% uranyl acetate. The samples were dehydrated in graded ethanol and embedded in epoxy resin. Ultrathin Sect. (60 nm) were prepared, stained with lead citrate, and finally imaged using a JEM-1400 electron microscope (JEOL LTD, Tokyo, Japan). For cell samples, pre-chill the cells in 2.5% glutaraldehyde for 1 hour, then scrape and centrifuge them into a pellet. Remove most of the supernatant, allow natural sedimentation for 1 hour, and then aspirate the remaining supernatant. Add 1 ml of pre-chilled 2.5% glutaraldehyde and fix at 4°C. Subsequent steps are similar to those for animal samples.

### Statistical analysis

All data were collected and analyzed in a blinded manner. Data were shown as mean ± SEM and analyzed using GraphPad Prism (GraphPad Software, Inc., La Jolla, CA, United States). Statistical analyses were conducted using two-tailed unpaired t-tests or one-way ANOVA followed by Tukey’s multiple comparisons test. A significance level of *p* < 0.05 was considered statistically significant.

## Electronic supplementary material

Below is the link to the electronic supplementary material.

Supplementary Material 1

## Data Availability

No datasets were generated or analysed during the current study.

## Abbreviations

Aβ: Beta-amyloid
AD: Alzheimer’s disease
AAV: Adeno-associated virus
CHX: Cycloheximide
GSK3B: Glycogen synthase kinase 3β
MAPT: Microtubule-associated protein tau
MWM: Morris water maze
MAPT: Microtubule-associated protein tau
NOR: Novel object recognition
PI3K: Phosphoinositide 3-kinase
mTOR: The mechanistic target of rapamycin
p-mTOR: phosphorylated mTOR
p-P70 S6K: phosphorylated P70 S6 Kinase
PI3K: Phosphoinositide 3-kinase
SNCA: α-synuclein
SYN: Synaptophysin
USP9: Ubiquitin specific peptidase 9
WT: Wild type
