# Supplementary material for: USP9X-mediated deubiquitination of Raptor contributes to autophagy impairment and memory deficits in P301S mice
Source: Cell Commun Signal. 2024 Oct 24;22:516. doi: 10.1186/s12964-024-01872-8 (PMC11515493; doi:10.1186/s12964-024-01872-8)
Supplement: Supplementary file 1 — Supplementary Material 1 [file 12964_2024_1872_MOESM1_ESM.docx]

**Supplementary figures 1 to 6**

**
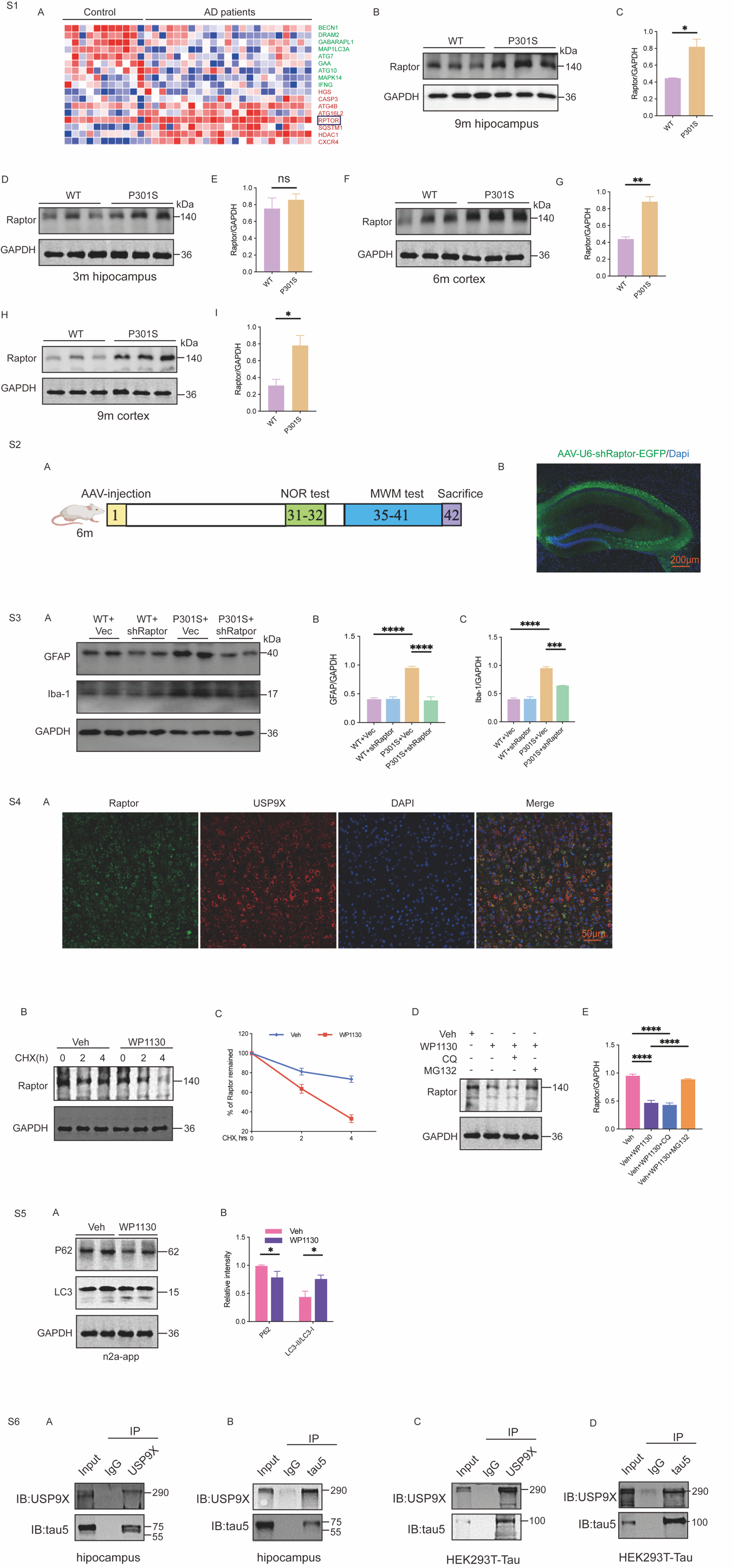
**

**Fig.S1** (A) A heat map depicting the abundance changes of differentially expressed proteins enriched in the autophagy pathway within the superior frontal gyrus of AD patients and controls from the GSE5281 dataset. Green indicates downregulated proteins, while red indicates upregulated proteins. (B, C) Representative western blots and quantification of Raptor protein levels in the hippocampus of 9-month-old P301S mice and WT littermates. n = 3 mice per group. (D, E) Representative western blots and quantification of Raptor protein levels in the hippocampus of 3-month-old P301S mice and WT littermates. n = 3 mice per group. (F, G) Representative western blots and quantification of Raptor protein levels in the cortex of 6-month-old P301S mice and WT littermates. n = 3 mice per group. (H, I) Representative western blots and quantification of Raptor protein levels in the cortex of 9-month-old P301S mice and WT littermates. n = 3 mice per group.

Data are presented as mean ± SEM. Unpaired t-tests were used to determine statistical significance. *p < 0.05, **p < 0.01, ***p < 0.001, ****p < 0.0001.


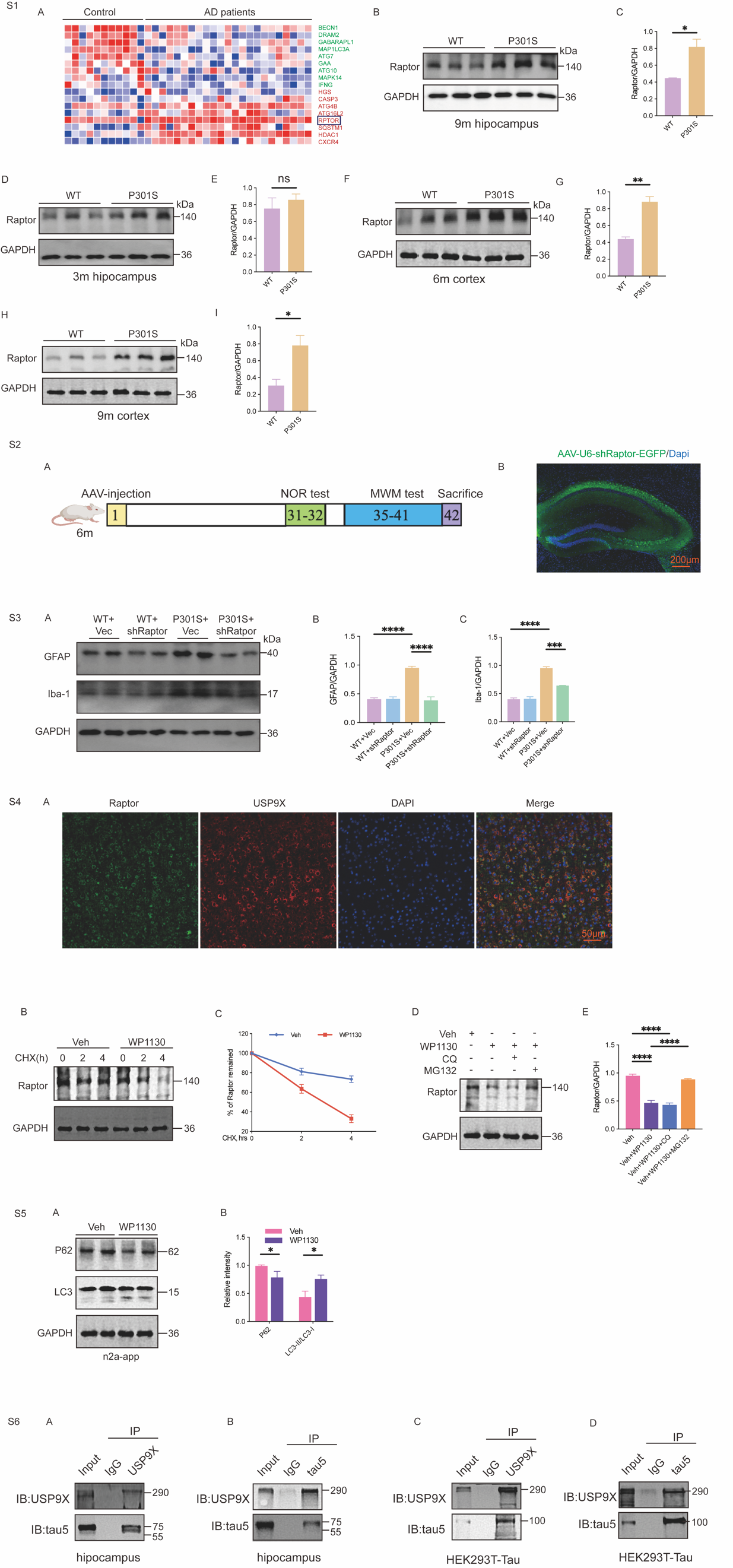


**Fig.S2** (A) Flowchart of the behavioral test. (B) Representative fluorescence image of AAV-U6-shRaptor-EGFP injection in the CA3 region of the mice hippocampus.


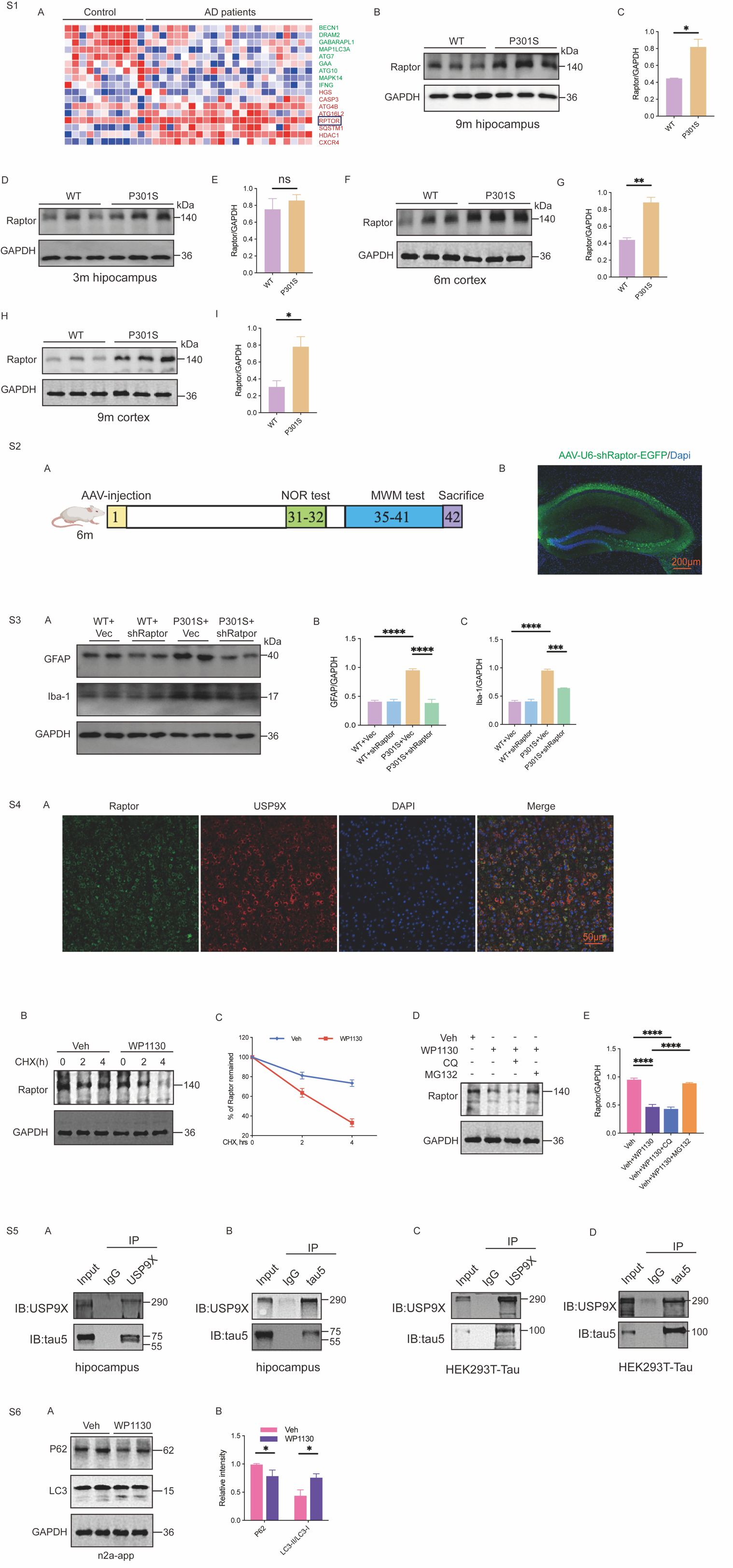


**Fig.S3** (A-C) Western blot analysis of GFAP and Iba-1 levels in mice and quantitative analysis. n = 3 mice per group. Data are presented as mean ± SEM. One-way ANOVA followed by Tukey's multiple comparisons test was used to determine statistical significance. *p < 0.05, **p < 0.01, ***p < 0.001, ****p < 0.0001.


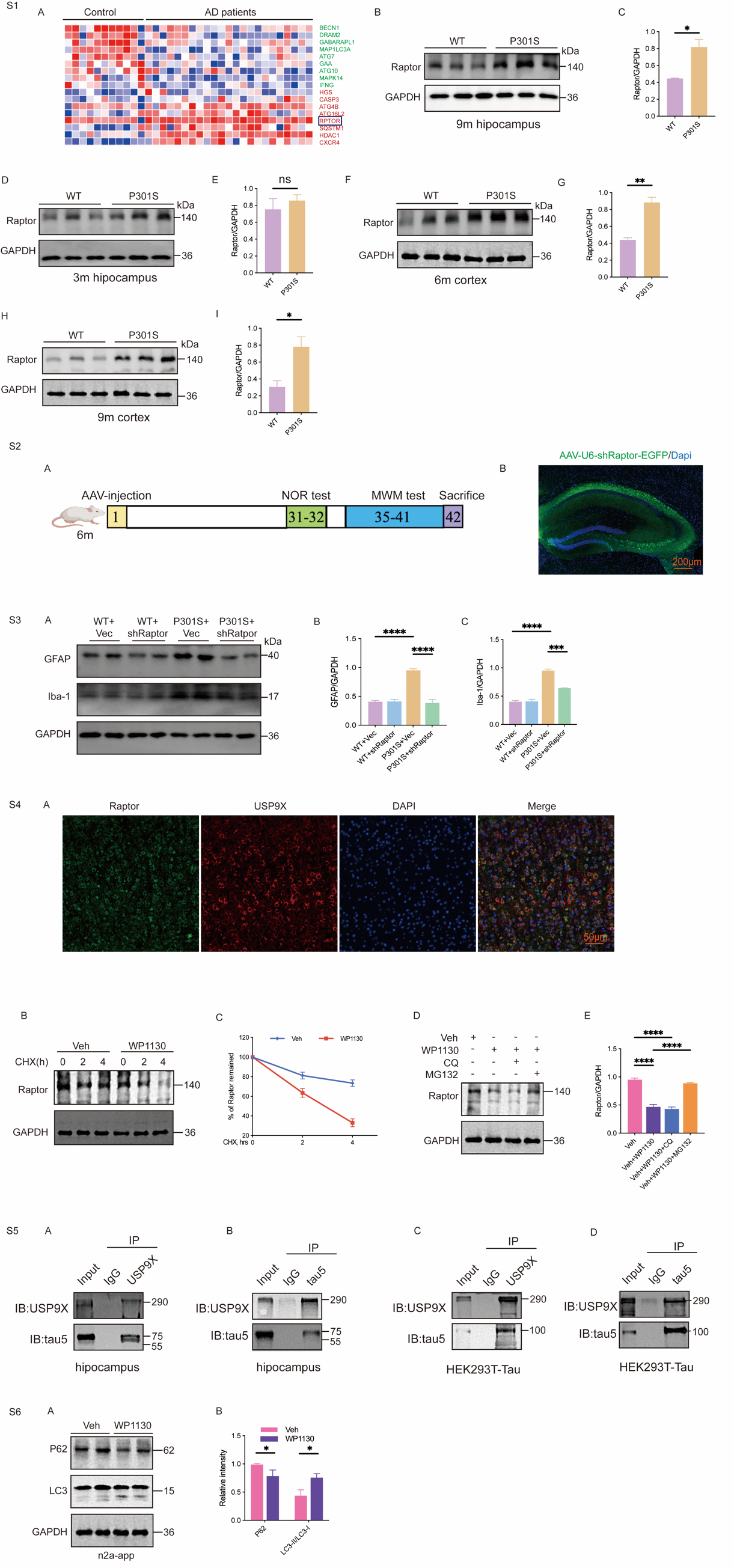


**Fig.S4** (A)Immunofluorescence staining of USP9X (red) and Raptor (green) in the cortex of 6-month-old P301S mice. Nuclei were counterstained with DAPI (blue). (B, C) HEK-293T cells were treated with 5 μM WP1130 for 4 hours, followed by CHX (20 μg/ml) treatment for the indicated times to inhibit protein synthesis. Protein levels were then analyzed by western blot and quantified. n = 3 per group. (D, E) In HEK-293T cells, treatment with 5 μM WP1130 for 6 hours significantly reduced Raptor levels. This reduction was reversed by MG132 treatment (20 μM) for 6 hours but not by CQ (20 μM) for 6 hours, as determined by western blot analysis. n = 3 per group.

One-way ANOVA followed by Tukey's multiple comparisons test was used to determine statistical significance. *p < 0.05, **p < 0.01, ***p < 0.001, ****p < 0.0001.


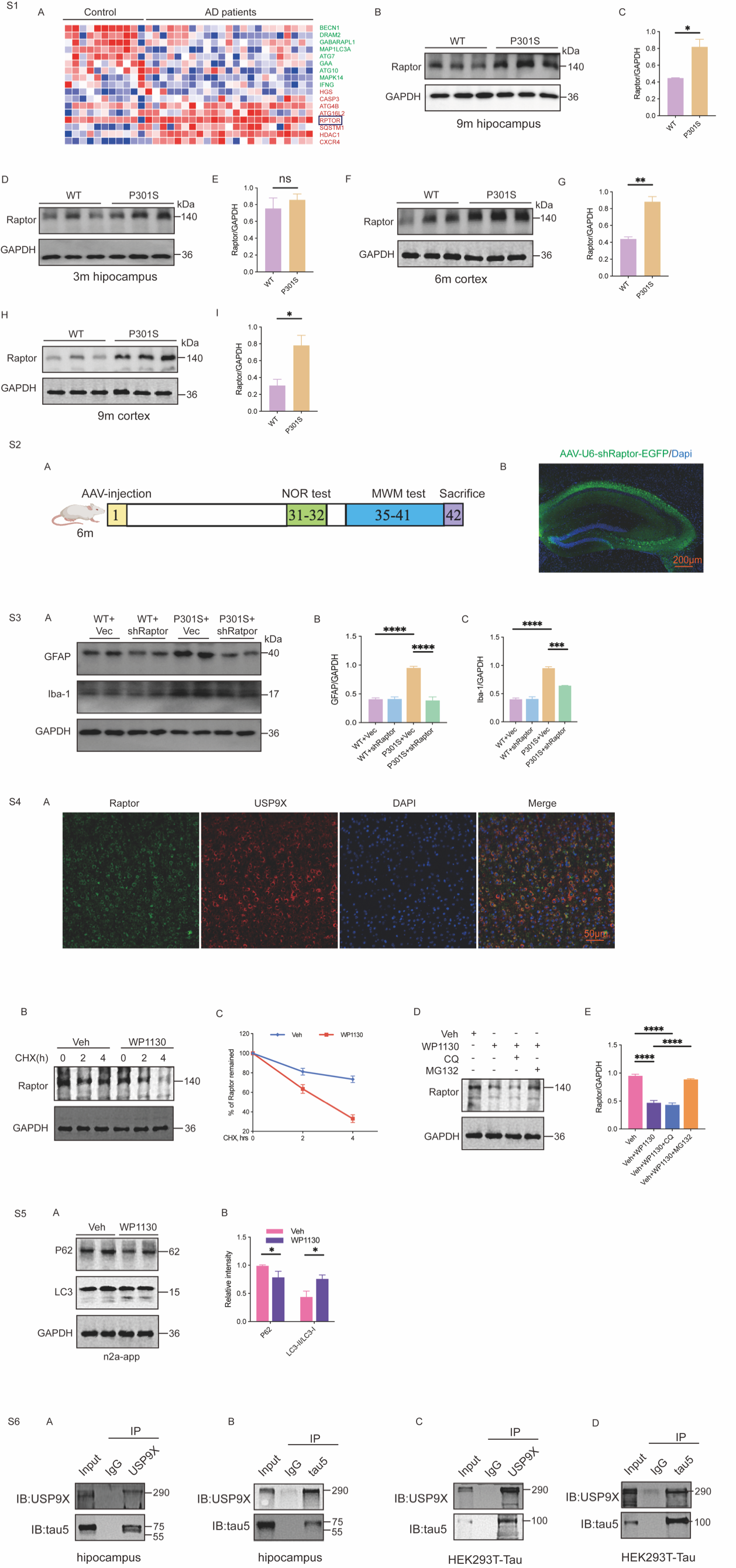


**Fig.S5** (A, B) n2a-app cells were treated with 5 μM WP1130 for 24 hours, followed by western blot analysis to detect P62 and LC3. n = 3 per group. Unpaired t-tests were used to determine statistical significance. *p < 0.05, **p < 0.01, ***p < 0.001, ****p < 0.0001.


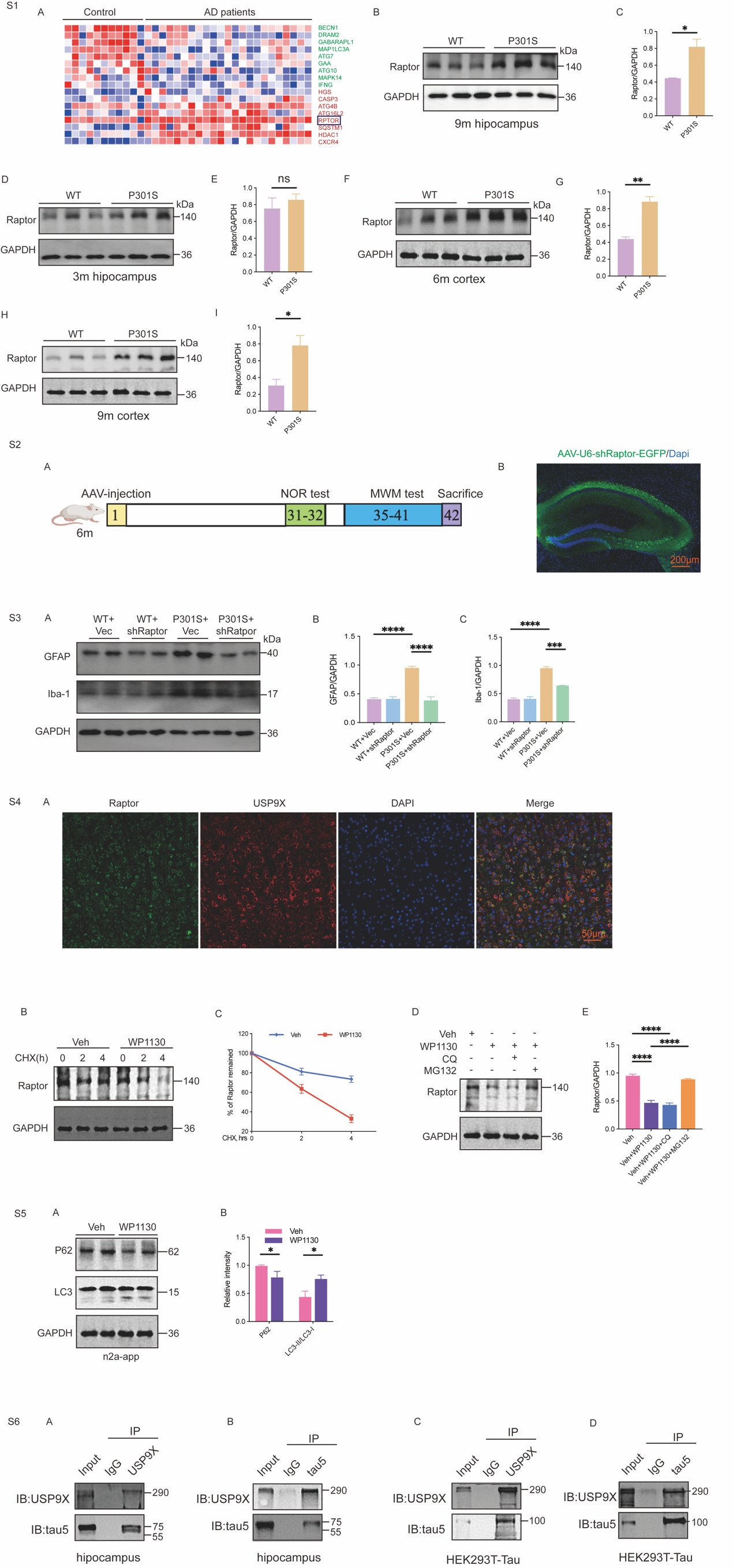


**Fig.S6** (A, B) Co-immunoprecipitation experiment demonstrating the interaction between USP9X and tau5 in the hippocampus of P301S mice. (C, D) Co-immunoprecipitation experiment demonstrating the interaction between USP9X and tau5 in the HE

Table S1. USP9X and autophagy

| **Cell Types** | **Processing Method** | **Results** | **Reference PMID** |
| --- | --- | --- | --- |
| FaDu cells | si-USP9X | p-P70S6K and P70S6K decreased | 27374971 |
| HeLa and 786-O cells | USP9X KO | P62 multi-site ubiquitination | 37260183 |
| C2C12 myoblasts | si-USP9X | p-P70S6K/P70S6K increased | 22544753 |
| ReNcell VM cells | sh-USP9X | p-P70S6K/P70S6K decreased | 28341829 |
| four types of pancreatic cancer cell lines | 0.625 μM WP1130，24h | Autophagy inhibition | **30118840** |
| CD8 T cells from CLP model liver tissue. | 30 mg/kg WP1130 | Autophagy activation | 36514222 |
| HEK293Tcells | si-USP9X | Beclin1 decreased | 25472497 |
| PDAC cells | miR-212 (inhibiting USP9X) | Autophagy activation | 30553134 |
| SH-SY5Y cells | si-USP9X | P62 shows no significant change | 22065755 |
| HEK293, HeLa, U2OS cells | 5 μM WP1130, 2h | ULK1 aggregation and autophagy inhibition | 26207339 |
